# Automated Growth Rate Measurement of the Facet Surfaces of Single Crystals of the β-Form of l-Glutamic Acid Using Machine Learning Image Processing

**DOI:** 10.1021/acs.cgd.3c01548

**Published:** 2024-04-05

**Authors:** Chen Jiang, Cai Y. Ma, Thomas A. Hazlehurst, Thomas P. Ilett, Alexander S. M. Jackson, David C. Hogg, Kevin J. Roberts

**Affiliations:** † Centre for the Digital Design of Drug Products, School of Chemical and Process Engineering, University of Leeds, Leeds LS2 9JT, U.K.; ‡ School of Computing, University of Leeds, Leeds LS2 9JT, U.K.

## Abstract

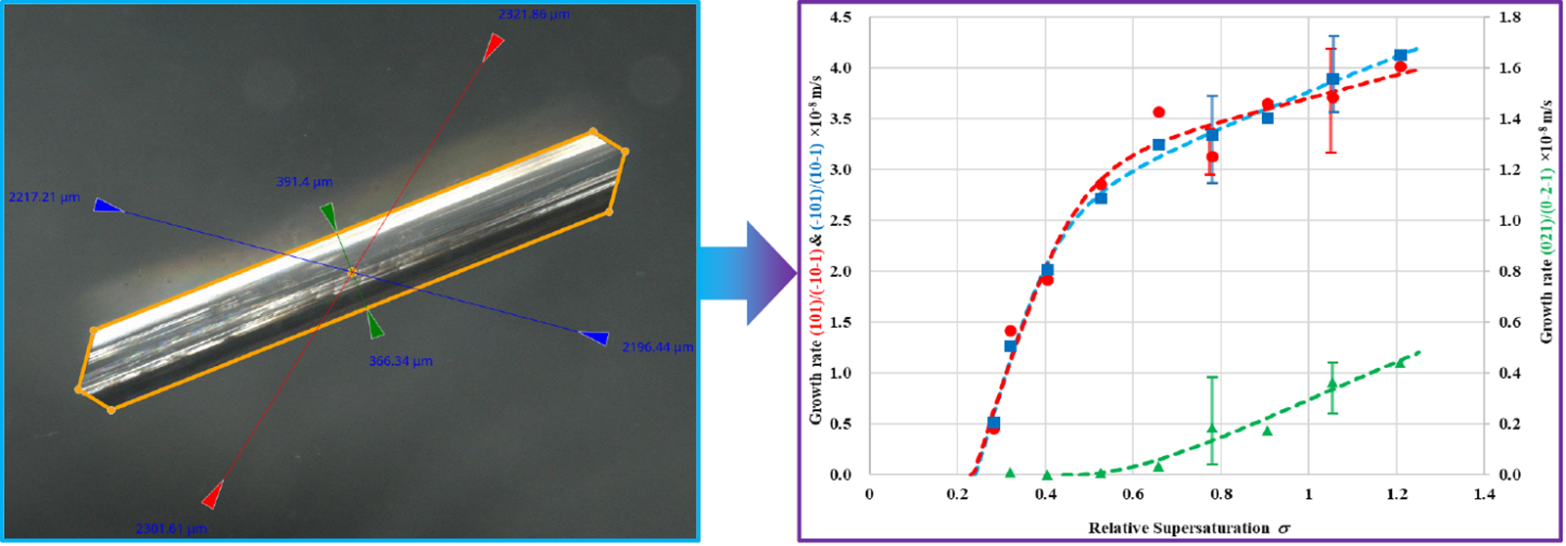

Precision measurement of the growth rate of individual single crystal facets (*hkl*) represents an important component in the design of industrial crystallization processes. Current approaches for crystal growth measurement using optical microscopy are labor intensive and prone to error. An automated process using state-of-the-art computer vision and machine learning to segment and measure the crystal images is presented. The accuracies and efficiencies of the new crystal sizing approach are evaluated against existing manual and semi-automatic methods, demonstrating equivalent accuracy but over a much shorter time, thereby enabling a more complete kinematic analysis of the overall crystallization process. This is applied to measure in situ the crystal growth rates and through this determining the associated kinetic mechanisms for the crystallization of β-form l-glutamic acid from the solution phase. Growth on the {101} capping faces is consistent with a Birth and Spread mechanism, in agreement with the literature, while the growth rate of the {021} prismatic faces, previously not available in the literature, is consistent with a Burton–Cabrera–Frank screw dislocation mechanism. At a typical supersaturation of σ = 0.78, the growth rate of the {101} capping faces (3.2 × 10^–8^ m s^–1^) is found to be 17 times that of the {021} prismatic faces (1.9 × 10^–9^ m s^–1^). Both capping and prismatic faces are found to have dead zones in their growth kinetic profiles, with the capping faces (*σ*_c_ = 0.23) being about half that of the prismatic faces (*σ*_c_ = 0.46). The importance of this overall approach as an integral component of the digital design of industrial crystallization processes is highlighted.

## Abstract 2

A new automated approach, using state-of-the-art computer vision and machine learning to segment and measure the crystal images, is developed for crystal sizing of β-form l-glutamic acid with high accuracy and fast processing time. Fast growth on {101} capping faces has a Birth and Spread mechanism with a dead zone of *σ*_c_ = 0.23, while the growth rate of {021} prismatic faces, previously not available in the literature, has a Burton−Cabrera−Frank screw dislocation mechanism with a dead zone of *σ*_c_ = 0.46.

## 1 Introduction

The manufacture of solid-form materials such as pharmaceuticals, foods, and agrochemicals often involves their processing in crystalline form with crystallization being used in order to isolate and purify high-quality products.^1^ Crystal size and shape and, through the latter, the corresponding surface chemistry can play an important role in the efficient performance of the downstream processes used for formulation such as filtration, drying, milling, blending, granulation, and tableting, with concomitant impact upon product performance. An in-depth understanding of crystal face-based growth as a function of the variation in the crystallization environment can thus be important in quantifying and hence controlling the crystallization processes. Within this perspective, the use of crystal size and shape measurements for determining crystal facet (*hkl*)-based growth kinetics for incorporation into morphological population balance process models^2,3^ and, through this, enabling the prediction and control of crystal size and shape represents an important element in digital crystallization process design.^4^

Molecular-based simulation approaches have been used to predict and understand crystal morphology associated with face-based crystal growth.^1^ However, while they provide a useful baseline, these techniques have not so far been able to predict the kinetic aspects of the growth process. Molecular modeling software such as HABIT98,^5^ for example, has been used to predict the crystal lattice energy, intermolecular interactions (synthons), relative growth rates, crystal morphology, etc. Joswiak et al.^6^ have used atomistic simulations to calculate kink site attachment rates for crystal growth rate prediction, while molecular dynamics (MD) simulations have been used to model the energetic balance between solute and solvent intermolecular interactions (e.g., references (7), (8)) and for investigating crystal nucleation and growth in the solution state.^9^ Despite this, current simulation approaches have not, as of yet, been found to be readily available to accurately predict de novo face-based crystal growth rates and their associated mechanisms under representative crystallization process conditions such as variations in solvent, temperature, solute concentration, and supersaturation.

Crystallization studies with online imaging systems have been performed to determine crystal growth kinetics within a population of crystals (e.g., references (10−17)), to investigate the effect of operating conditions on the processing behavior and to monitor variations of crystal size and shape during the processes (e.g., references (18−20)). However, the crystal images captured from within a crystallizer are, by their very nature, transient due to their motion under process hydrodynamic conditions. As a result, often such images can be quite poorly resolved due to the rotation of crystals under agitation making it quite challenging to track the growth behavior of individual crystals. In comparison, single crystal growth measurements using temperature-controlled growth cells together with high-resolution optical microscopy provide a much more effective and accurate approach for facet growth measurements, e.g., references (15,21−25), when compared to other methods such as atomic force microscopy,^26^ rotating disk techniques,^27^ or microfluidics.^28^

A growth cell with temperature control by a recirculation bath has been developed to grow single crystals from solution using a microscope to capture images of the growing crystals for growth rate determination along individual face directions. This has been previously reported for studies of ibuprofen,^22,23,29^ methyl stearate,^25^*para*-aminobenzoic acid,^8^ LGA,^14,21,30^ α-glycine,^24^ and tolfenamic acid.^31^ Traditionally, manual image analysis methods have been used for processing the images, but these can be problematic due to their low accuracy and consistency due to human error. This approach can also be highly time-consuming with concomitant impact upon the operator’s health from continuously interacting with a video screen and tracking the dynamic progression of the crystal edges during the growth process. The Hough transform method has been used to find lines in very ideal crystal images captured from a growth flow cell for measuring facet growth rates of α-glycine.^24^ However, these techniques could often not provide accurate enough outputs for less ideal images, as this technique was not always able to provide clean lines or too many lines for the analysis algorithm to work consistently.

Analysis of crystal growth kinetics and mechanism has been based on growth interfacial models such as BCF,^32^ B&S,^33^ and power law^34^ and, in the latter case, where the exponent is 1, then this would correspond to an unstable rough interface growth.^35^ Such growth interface kinetic models have been integrated to incorporate the effect of solute mass transfer^25,36^ on crystal growth mechanism, using user-defined functions such as developed by Camacho et al.^25^ to fit the growth data. Through this approach, the effects of the incorporation of growth units into the crystal surface (growth surface integration, GSI) and their diffusion within the solution (mass transfer, MT) on facet crystal growth kinetics and mechanisms can be assessed and quantified with respect to which of these two kinetic aspects is the rate-limiting step. Apart from the interfacial kinetics models used to model the GSI process during crystal growth, MT may also have a significant impact on the facet growth rate.^25^ This has been previously highlighted by Garside and Tavare^37^ through their definition of an effectiveness factor and their highlighting of the importance of growth rate dispersion for modeling its influence on crystal size distribution in a crystallizer. An improved understanding of MT has been demonstrated by Nicholson et al.^38^ who used laser interferometry to characterize the MT within the boundary layer between the bulk solution and the crystal faces during growth and dissolution.^39^

Attempts have been made to utilize deep learning technology to track and measure individual crystals in images captured during solution crystallization processes.^20,40^ Crystal properties including size distribution, morphology, and surface area of LGA α and β polymorphs were obtained in situ for individual crystals with the image processing speed achieving up to 10 frames per second.^20^ Bischoff et al.^40^ developed software to generate a synthetic data set of realistic protein crystal images in suspension by employing ray tracing rendering algorithms for use in supervised machine learning. The robust object detection models developed using this data set were used to quantify and characterize protein crystallization processes under different conditions, especially with low-resolution imaging systems. However, the use of machine learning techniques for characterizing facet crystal growth has, up to now, been quite limited.

Significant advancements have been made in the realm of image segmentation by using deep learning models. Modern image segmentation tools, such as Mask-RCNN,^41^ have been found to exhibit high accuracy and efficiency in segmentation outcomes but require customization and additional training for specific image domains. Consequently, segmentation models with the ability to generalize across various tasks hold a distinct advantage, especially when additional labeled data have not been readily available. Currently, the state-of-the-art approach in this field is the segment anything model^42^ (SAM) from Meta AI research. SAM is particularly noteworthy for its remarkable ability to segment diverse objects in any domain. This prowess is attributed to its training on an extensive data set of over 11 million images, encompassing one billion segmentation masks.^42^ The capability to identify virtually any object within an image establishes a promising foundation for the development of a tool tailored to detecting crystals in microscope images.

This study focuses on the crystallization of l-glutamic acid (LGA), an amino acid widely used in the food and pharmaceutical industries. LGA has been also widely used as a model compound in crystallization process research^10−16,19−21,43−45^ and has two polymorphs: the prismatic-shaped metastable α-form and the more needle-like shaped stable β-form.^45^ Data on the needle-like β-form LGA (β-LGA) is widely available, and many studies have investigated its crystallization behavior and processing performance. Previous work has examined the growth rate measurements of LGA crystallization from solution using instruments such as *FBRM*(10,46) and laser light scattering^44^ using a spherical crystal assumption in the latter case even for the needle-like β-LGA, hence only a one-dimensional (1D), spherical-based growth rate could be measured. With the advances of in-process imaging systems, online images of LGA crystals captured during crystallization processes have been used to estimate two-dimensional (2D, length and width) growth rates (e.g., references (11−16)). However, the growth rates reported for the published slow growth face in the width direction of β-LGA have been found to be highly variable with high variance^16^ or indeed could not be realistically obtained.^30^ Kitamura and Ishizu^21,30^ measured the growth rates of α- and β-LGA crystals and found that the growth of the prismatic faces of β-LGA was too slow to produce meaningful growth rate/kinetics, which echoes the large discrepancy of this face’s growth rate when obtained from in-process crystal images in crystallizers.^16^ Previous studies also revealed that the single crystal growth rates determined under stagnant conditions are comparable to those estimated from within crystal populations in agitated vessels,^15,23^ indicating the value of obtaining accurate growth rates under more easily controlled stagnant environments. The crystal shape of LGA has been investigated by Turner et al.^47^ who developed a digital mechanistic workflow for predicting crystal morphology of LGA in aqueous solution revealing the β-LGA structure to have an anisotropic distribution of its dominant extrinsic synthons across its crystal habit faces, directly impacting upon the relative crystal growth rates of the β-LGA faces and rationalizing the understanding of its needle-like crystal morphology.

In this paper, crystal growth rates of the capping and prismatic faces of the β-form l-glutamic acid have been investigated. A machine learning powered automatic analysis tool has been developed that demonstrates improved accuracy and efficiency over manual and semi-automatic measurement approaches. Using this approach, accurate, manually validated measurements of face-based growth rates and growth mechanisms for β-LGA were obtained.

## 2 Materials and Experimental Methods

### 2.1 Materials

l-Glutamic acid (LGA) with a purity of ≥99% was purchased from Sigma-Aldrich. The distilled water was obtained in-house. The LGA was directly used for this study without any further purification.

**Figure 1:**
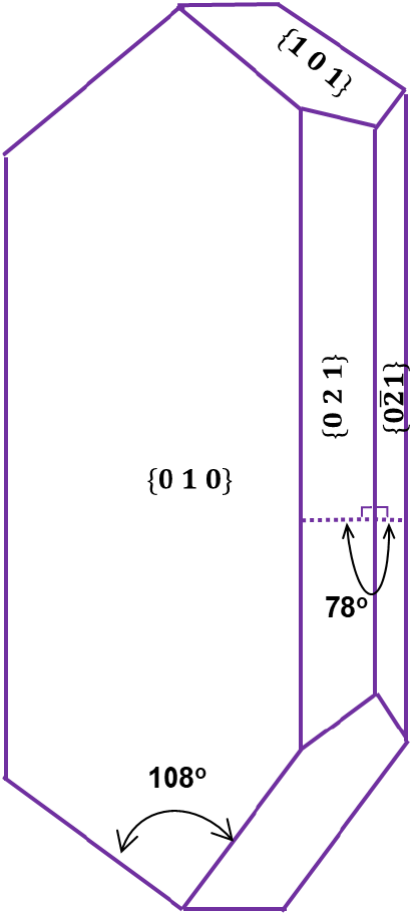
Schematic of the β-LGA crystal morphology together with some face-to-face angles.

This work focused on the faceted crystal growth of the stable β-form LGA; hence, no polymorphic transformation issues were present. Based upon the crystal structure for the β-form of LGA,^54^ the schematic crystal morphology is shown in Figure 1. Note that the crystallographic setting of the unit cell parameters (*a*, *b*, *c*) adopted here is *a* < *c* < *b* after the convention by Davey et al.^54^ (LGLUAC11) which is different from that used by Turner et al.^47^ (LGLUAC01) which used *a* < *b* < *c*. In the former case, the angles between (101) and (10–1) faces and between (021) and (02–1) faces are 108° and 78°, respectively.^54^

### 2.2 Experimental Apparatus

**Figure 2:**
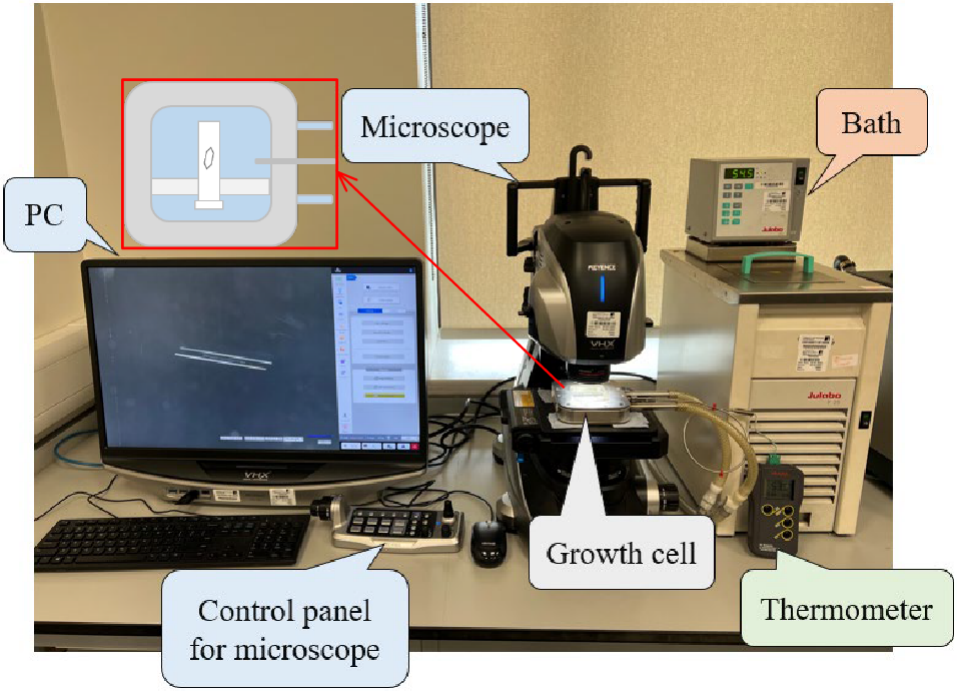
Experimental setup for the growth rate measurements of β-LGA single crystals in individual face directions. A single crystal seed is placed in the growth cell and maintained at the target temperature by a recirculating water bath. A digital microscope records images periodically on the computer.

A temperature-controlled crystal growth cell system (Figure 2) to capture high-quality single crystal images of β-LGA comprises a glass cuvette cell,^22,25^ a Keyence VHX7000 digital optical microscope^48^ integrated with 3 zooming lenses (20–100× , 100–500×, and 500–2500×) and a 1/1.7-in. 4K CMOS image sensor (108 megapixels) camera, connected to a computer with image capturing and analysis software. The crystallization vessel itself was a UV cuvette glass cell with a volume of 0.5 mL (sizes of 54 × 10 × 1 mm) submerged in a small shallow cell filled with circulating water whose temperature was directly controlled by a Julabo F25 recirculation bath (Figure 2).

### 2.3 Experimental Methods

#### 2.3.1 Preparation of β-LGA Seeds

All experiments in this study were carried out using crystal seeds of LGA β-form single crystals, which were prepared by slow evaporation from solution with 10 g of LGA and 1 L of water and corresponding to a saturated temperature of around 33 °C. The solution was heated to ensure complete dissolution and then left in a crystallizing dish covered by parafilm, which was pierced in several places to allow for slow evaporation and left at room temperature for several days resulting in the formation of crystal seeds. To minimize the potential effect of seed size on growth rate, crystals with similar lengths were selected for use as seeds, and these were then used to measure the growth rates under different supersaturations.

#### 2.3.2 In Situ Growth Rate Measurements

The solubility of β-LGA in distilled water has been previously reported in the literature,^49^ and this was used for this study. The LGA solution for all experiments was prepared by dissolving 35 g of the solute LGA in 1 L of deionized water (corresponding to a saturation temperature of 67 °C). The solution prepared was transferred into the cuvette cell using a pipet. The crystal seeds of β-LGA were placed into the cuvette cell, which was then rapidly sealed and carefully and firmly attached to the bottom of the growth cell. After this, the growth cell was sealed within its water bath at a preset initial temperature of 67 °C in order to limit potential secondary nucleation. When a β-LGA seed was placed in the growth cell, it was arranged that the slowest growth face {010}, hence, the surface with the largest surface area, would lay flat on the cuvette base. This geometry allowed measurement of the growth of other faces {101} and {021} through captured 2D crystal images. Hence the growth of the individual growth directions normal to the {101}, {10–1} and {021} habit planes under different solution supersaturations could be measured within the cuvette. The bath temperature was then set to a value higher than the saturated one (67 °C) to slightly dissolve back the surfaces of the single crystal seeds in order to attain the required seed size and also to remove any possible surface imperfections. The solutions were then cooled down to a constant temperature between 44 and 60 °C (corresponding relative supersaturations σ ranging from 1.21 to 0.28 with σ being defined as a ratio of the difference between solute concentration and its solubility at the same temperature to the solubility) until the end of the growth process, hence achieving the solution supersaturation at a specific value. Note that all experiments performed in this study were conducted within the metastable zone; under conditions where no further crystal nucleation (primary or secondary) took place. Furthermore, the whole internal area of the cuvette including its corners/edges and also seed surface was checked using the microscope at a higher magnification by scanning its translation stage to ensure only a single crystal seed existed in the cuvette before starting automatic image recording of faceted crystal growth.

Temporal images were captured under reflective light mode with automatic focus at constant time intervals during the entire crystal growth process and saved with the associated time, lens information and scale bar for reference. The preset time intervals were typically in a range of 1–10 min depending on the size of a seed (length = 1600–1800 μm and width = 300–1220 μm), temperature (44, 46, 48, 50, 52, 54.5, 57, 59, and 60 °C), and the corresponding relative supersaturation (σ = 1.21, 1.05, 0.91, 0.78, 0.66, 0.53, 0.40, 0.32, and 0.28). Note that the facet growth rate was calculated from the projected distances of the habit faces by allowing for the appropriate interplanar angles. Experiments at relative supersaturations of 0.78 and 1.05 were repeated six and five times, respectively, to verify the repeatability of growth rate measurements. Additionally, the distances calculated were based on the normal distance of a face from the nucleation center of the crystal, i.e., half of the measured distances between paired faces.

## 3 Data Analysis

### 3.1 An Automated Method for Crystal Growth Measurement

To evaluate the feasibility of an automated method for crystal growth measurement, a fully automatic method based on machine learning and two baseline methods (manual and semi-automatic) were applied to the same set of crystal images. These images were captured under the same relative supersaturation of 1.05, yielding a data set of 235 images. All images were processed by using the automatic method. For comparison, a subset of 21 images were selected and processed with the manual and semi-automatic measurement methods.

#### 3.1.1 Baseline Methods

**Figure 3:**
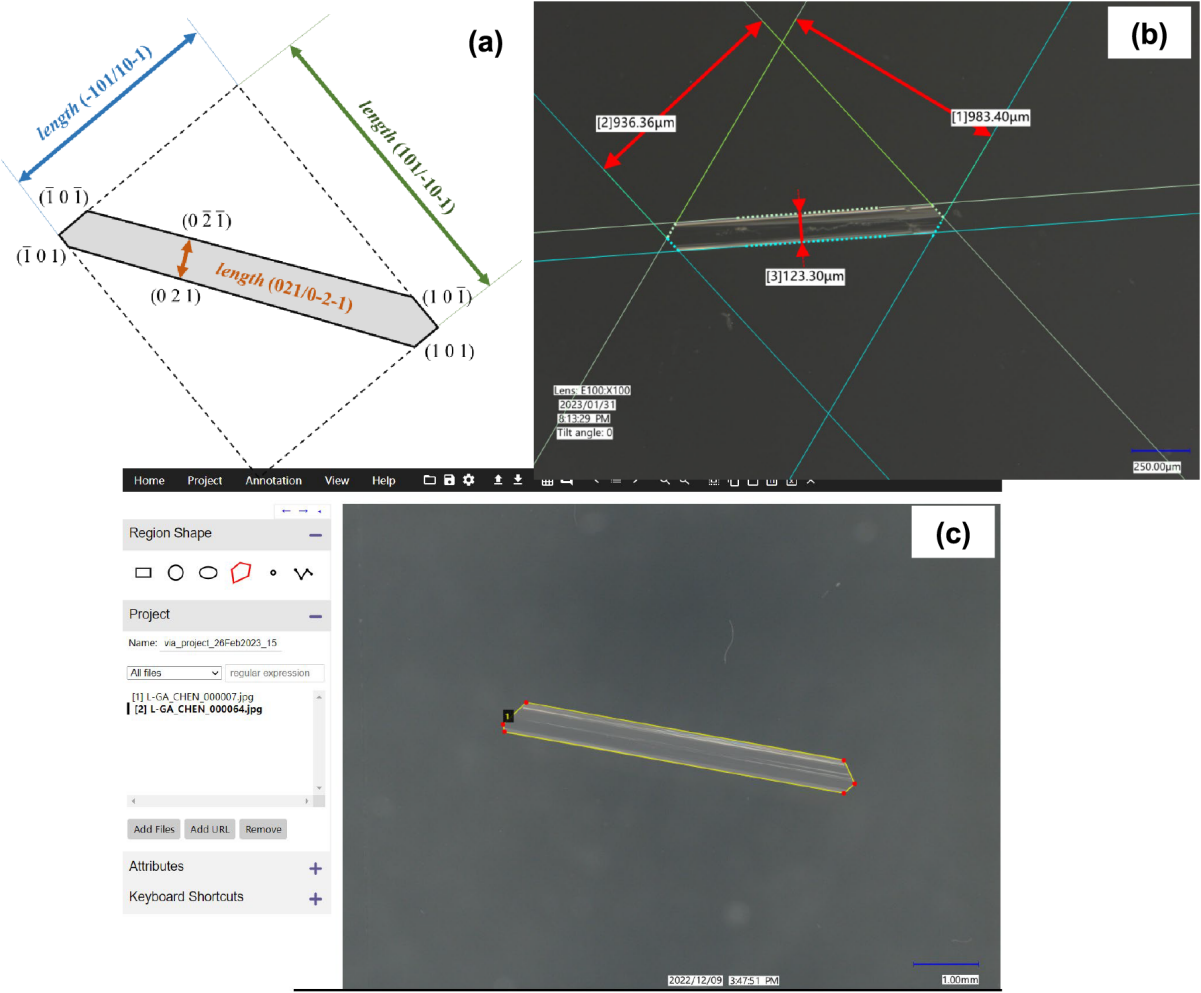
(a) Facet growth is determined by measuring the distances between opposing parallel crystal faces; (b) normal distances measured using Keyence software for the paired faces of β-LGA single crystal (manual method); (c) user interface of VGG Image Annotator for labeling coordinate positions (semi-automatic method).

In this study, several pairs of opposite faces of the β-LGA crystal were selected for growth rate investigation (as shown in Figure 3). The Keyence measurement software^48^ was used to process the crystal images captured by manually drawing parallel lines along the edges of two paired crystal faces on a crystal image, hence determining the normal distance in the pixelated image between these two lines. Note that the normal distance between the paired (021)/(0–2–1) faces was calculated based on the projected width and the angle between (021) and (02–1) faces. Further details can be found in Section S3. The actual distance in length units was found based on the calibrated actual pixel size. Through this, the actual distances between paired faces were directly obtained (Figure 3). The procedure was repeated for all paired faces and all crystal images at various supersaturation levels. These distances were then used in the fitting with respect to time used to calculate the face-based growth rates.

A semi-automatic measurement method was developed with an OpenCV-Python code in which users provided the coordinates of six vertices identified from the 2D projected crystal contour of β-LGA. A third-party UI tool, VGG Image Annotator,^50^ was adapted for labeling the coordinate’s positions (Figure 3). The code automatically measured the corresponding real normal distances based on these coordinates and then saved these distances with associated time when capturing the image for crystal facet growth rates and kinetics.

#### 3.1.2 Automatic Method

**Figure 4:**
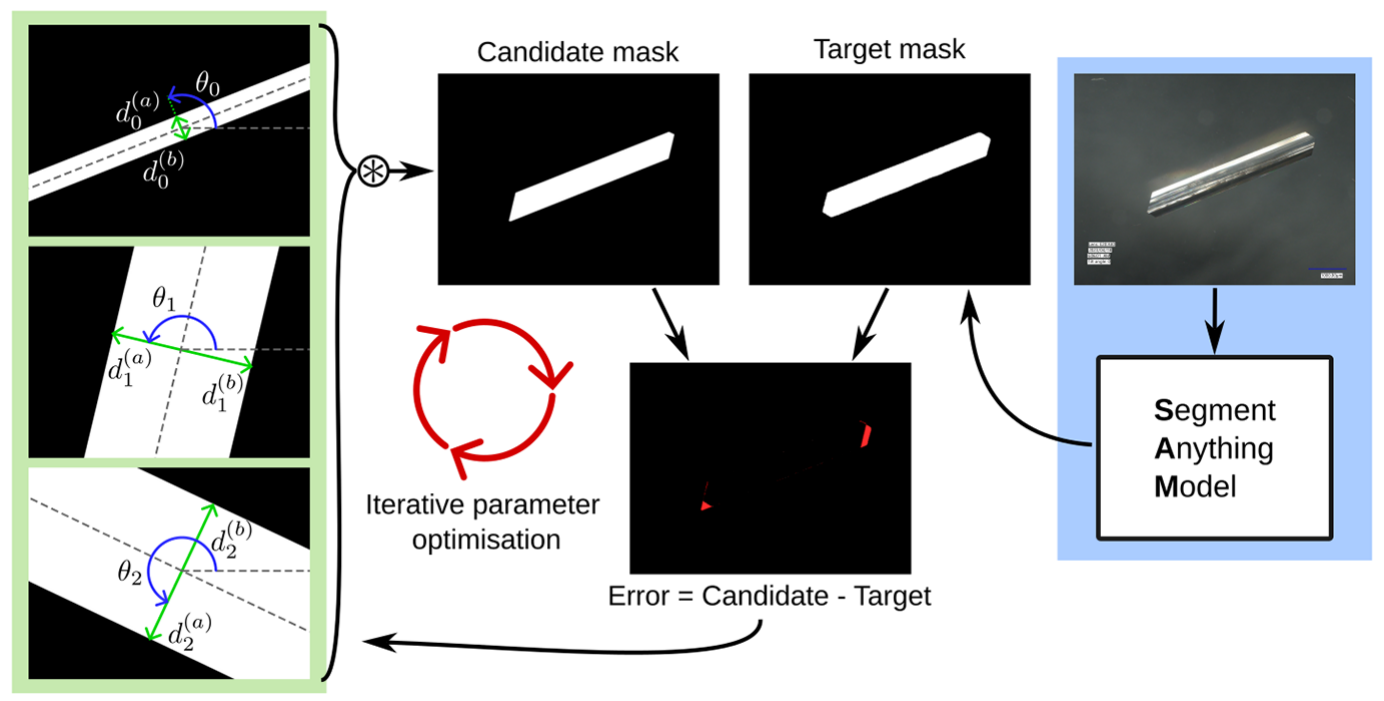
A candidate hexagon shape is constructed from the intersection of three sets of parallel lines. The generated candidate mask was compared against a segmented mask of the input image as produced by SAM. The error (the difference between the masks) was used to iteratively update the parameters by gradient descent.

The automatic crystal sizing process encompassed two distinct stages: (1) segmentation of the images to separate the crystals from the background and then (2) fitting to the β-LGA’s characteristic hexagon morphological shape to match the segmentation masks from stage (1). The overall process is outlined in Figure 4.

The segmentation stage was carried out using the state-of-the-art segment anything model (SAM), developed by MetaAI.^42^ SAM consists of a neural network that takes an image and approximate coordinates of a point of interest and returns a binary mask the same size as the input image, where values of 1 correspond to the foreground object and values of 0 correspond to the background. This is an off-the-shelf, open-source tool that requires no additional data, no training, and no further parameter tuning to produce very effective segmentation results. SAM provides an effective and robust method for directly producing crystal silhouettes from the images.

In the second stage, hexagon shapes were fitted to the segmentation masks. This is a direct optimization approach that requires no separate training and no data set other than the crystal images being analyzed. These shapes were parametrized by a center point, three angles, and three pairs of lengths. Each angle and distance-pair defined two parallel lines that, after optimization, aligned to a pair of parallel faces in the crystal image. The three sets were used to construct three bounding region masks (i.e., binary images), and a final hexagon shape was produced by taking the product of these masks (Figure 4). The first angle defines the perpendicular direction, corresponding to the shortest face distance. The second and third angles are manually defined relative angles to be added to the first angle. The relative angles could be estimated by hand from an image or, as in the case of β -LGA, looked up from the theory^16,21^ with an angle of 108° between the two capping faces (Figure 1) and 126° between the capping and prismatic faces being taken. A distance-pair was measured from the origin in each direction and by allowing these to differ from each other, they could capture asymmetric crystal growth and even continue to track distances when faces have “grown out”. Note that this latter case could result in a fitted shape with fewer than six sides.

The fitting process was performed by using iterative gradient descent. A batch of initial candidate hexagons were parametrized, their corresponding masks generated, and an error was calculated as the mean squared pixel-difference between the masks of the input crystals (pregenerated by SAM) and the masks for the candidate hexagons. Additional regularization loss terms were also added: the sum of the distances (to ensure a minimal bounding polygon), the sum of squared differences between the distance-pairs (to encourage some symmetry), and a temporal loss term to ensure the distances change smoothly across the sequence.

As the generation of candidate masks from the hexagon parameters used differentiable operations, gradients with respect to the parameters could be calculated by automatic differentiation using PyTorch^51^ and parameters were updated using a gradient descent optimization algorithm (Adam^52^ was used for this, but others were similarly successful). The optimization was run until convergence with the learning rate decreasing exponentially as the loss plateaus to ensure stable and smooth results. Fitting a typical sequence of 250 images was found typically to take about 20 min on a V100 GPU machine (∼5 min for SAM and ∼15 min for hexagon fitting).

**Figure 5:**
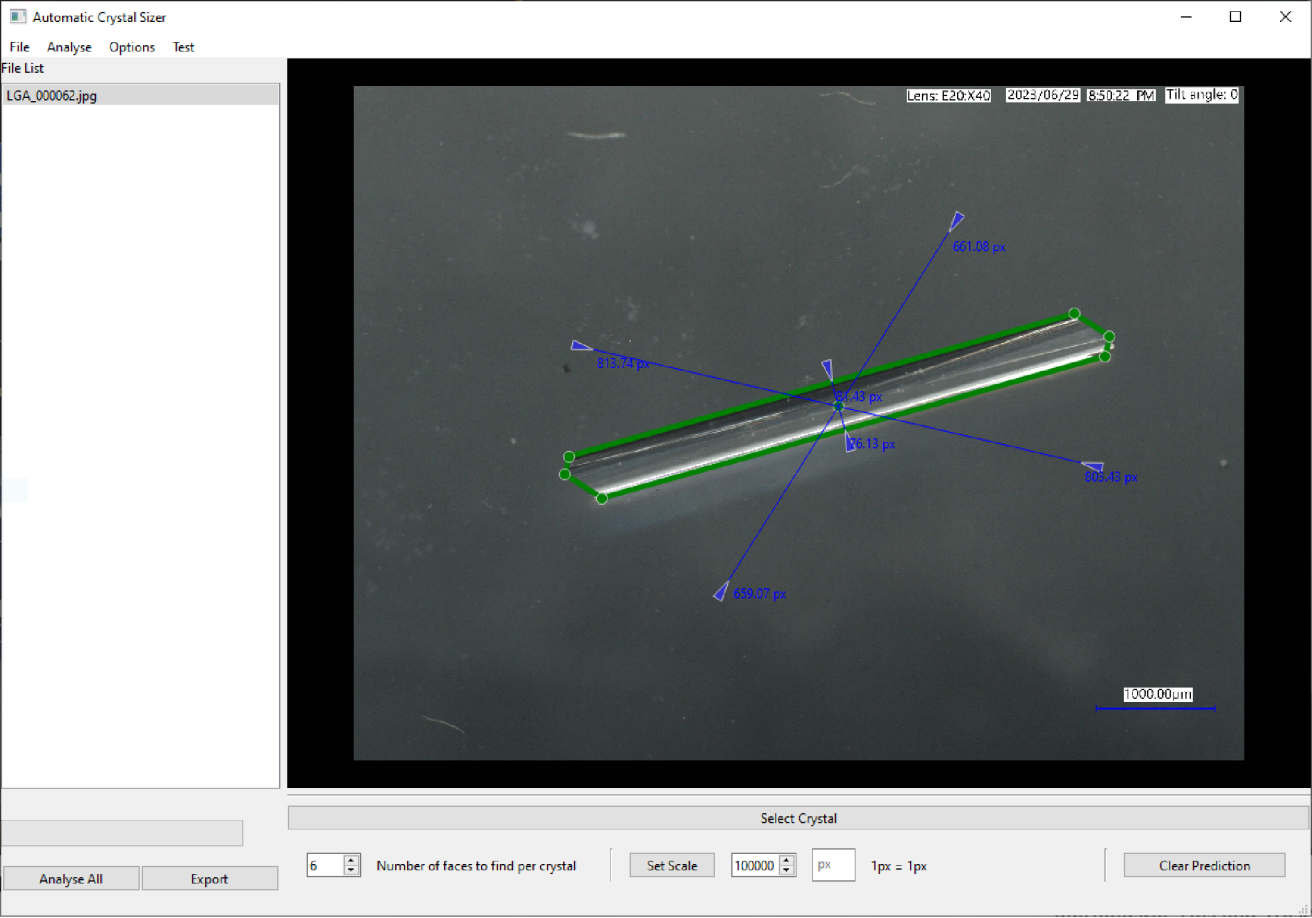
A screenshot of the Automatic Crystal Sizer. A crystal had been selected in the image and automatically measured. The green lines indicate the boundary of the crystal found by the software, and the blue arrows show the measured perpendicular distance from the center of the crystal to each crystal edge.

This process has been developed into a software package known as *Automatic Crystal Sizer,* which performs the overall task automatically once the user has selected the desired crystal to be measured. In Figure 5, a screenshot of the software is shown in which a crystal image was selected and automatically measured. The green lines indicate the boundary of the crystal found by the software, and the blue arrows show the measured perpendicular distance from the center of the crystal to each crystal edge. The scale is set by typing in the units of the scale in the image and then selecting it. A sequence of images, from a time series of microscope images observing the growth of a crystal over time, can be batch processed without intervention. By selecting a crystal in any image of the sequence, the software tracks the crystal forward and backward in time automatically, using the center of the crystal in the previous image as its new point of interest. Once completed, the measurements were exported to a *Microsoft Excel* file, where each image is listed with its associated hexagon parameters. These values can then be used for growth rate measurements.

### 3.2 Analysis of Crystal Growth Rates and Derivation of Growth Interface Kinetics

The crystal growth kinetic mechanisms including effects of MT and GSI were assessed with respect to establishing mechanistic models^25^ by
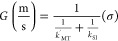
1where *G* is the facet growth rate; σ is the relative supersaturation;  is the resistance of GSI; and  is the MT resistance, satisfying
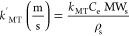
2where ρ_s_ is the solute density, MW_s_ is the solute molecular weight, and *C*_e_ is the equilibrium concentration (solubility).

For the LGA examined in this study, MW_s_ = 147.13 g mol^–1^ and ρ_s_ = 1.54 g cm^–3^. *C*_e_ can be calculated using the original solution concentration *C*_0_ = 237.88 mol m^–3^ and relative supersaturation during growth:
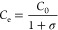
3

In eq 1, the *k*_SI_ term can be modeled based upon the three standard mechanistic models:^1^

power law model,
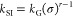
4

B&S model,
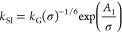
5

BCF model,
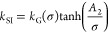
6where  is the growth rate constant, *r* is the growth exponent, and *A*_1_ and *A*_2_ are thermodynamic parameters. The situation where *r* = 1 corresponds to a rough interface growth mechanism.

The facet growth rates at supersaturations of 0.78 and 1.05 with six and five repeats, respectively, were further processed to find their mean values with the corresponding standard deviations. Further details can be found in Section S4. The mean growth rates were used for the determination of the growth kinetic mechanisms.

Comparison between the fitted parameters with respect to eq 1 was used to assess whether the growth rate was limited by either the MT or GSI.

The experimental fits to the observed data were used to estimate the face-specific critical supersaturation *σ*_c_ for growth, i.e., when *G* = 0 and setting *σ*_c_ equal to σ.

The data fitting to the models was carried out using Origin software^53^ with the Levenberg–Marquardt algorithm with the fitting of the data assessed through *R*^2^ (coefficient of determination) being used to assess the regression quality.

For comparison purposes, the measured growth rates in the length and width directions of β-LGA crystals from the literature were converted to the facet growth rates for the {101} capping and {021} prismatic faces using the corresponding angles of 108° and 78° (Figure 1), respectively.

## 4 Results and Discussion

### 4.1 Comparison Between Baseline and Automatic Methods

Analysis of the growing sizes of the β-LGA {021}, {101}. and {10–1} faces revealed that at the early stage of the growth process, there was a good linear fit between the paired interplanar projected distances with time as evidenced by an *R*^2^ of 0.99 (Figure S1), consistent with only a slight consumption of the solute, hence the initial supersaturation and with no obvious evidence for growth rate dispersion.^3,37^ However, at a later stage, the measured growth rates were found to deviate significantly from a linear relationship reflecting the depletion of the solute. Therefore, the slopes of the fitted linear equations using the early stage data were used as a measure of the growth rates of the paired faces at a given solution temperature or supersaturation. Figure S1 provides further information about the linear fitting for the determination of the growth rate.

**Figure 6:**
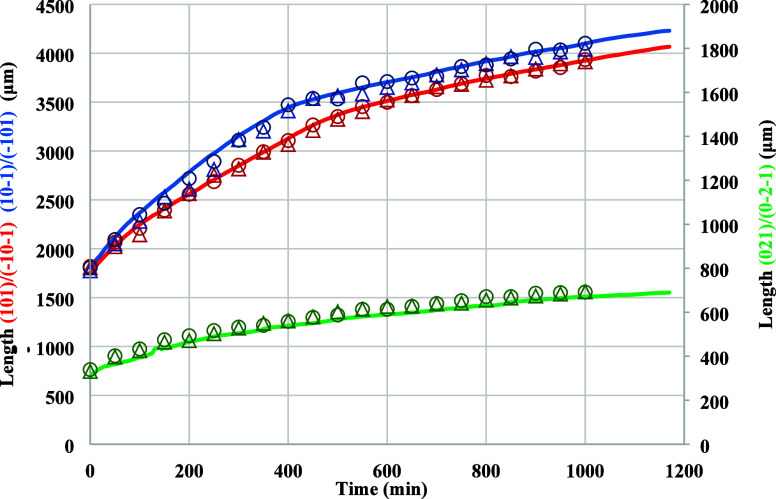
Comparison of the normal distances (lengths) between paired crystal faces during crystal growth in a growth cell, using the manual (open triangle), semi-automatic (open circle),and automatic sizing (line) methods (red: (101)/(−10–1) faces; blue: (10–1)/(−101) faces; green: (021)/(0–2–1) faces). The images were captured under a relative supersaturation of 1.05 (run #1, Section S4).

A comparison between the results of the crystal length measurements based on the three analytical methods is given in Figure 6. The analysis of these results reveals very close agreement with a discrepancy of ±5%. Taking the manual measurement as the ground truth, the average relative percentage errors of the other two methods are both under 5%, which is within an acceptable margin of error. An estimate of the image processing times reveals that they are typically approximately 114, 57, and 5 s per image, respectively, highlighting the advantages of the automatic sizing method for delivering more reliable and consistent results over a much shorter data processing time. Hence, the automated method allows users the ability to process a much wider range of in situ data sets of temporal crystal images rather than having to rely upon the analysis of only a selection of representative images using either the manual or semi-automatic methods. Reflecting upon this outcome, the subsequent analysis of all further results presented here were obtained using the automatic sizing approach for efficiently, accurately, and consistently measuring the facet growth rates.

### 4.2 Face-Specific Crystal Growth Rates as a Function of Solution Supersaturation

The results of the crystallization experiments carried out spanning nine different solution supersaturations are summarized in Figure S6. These contain, respectively, a typical sequence of the images and typical facet growth distances, i.e., the normal distance (length of paired faces) in a face direction. At two representative supersaturations (σ = 0.78 and σ = 1.05), the repeatability of the experimental results were checked by six and five repeated runs, respectively, as shown in Figures S4 and S5, revealing standard deviations (Tables S2 and S3) that are consistent with literature data.^22^ Further details can be found in Section S4. The measured crystal facet lengths, as a function of time, for all nine supersaturations are given in Figure S6. Linear fits to the early stage of the length data with time (Section 4.1) revealed the facet crystal growth rates with a typical fitting result, which is plotted in Figure S1.

Table 1 lists the face-base growth rates of the capping faces (101)/(−10–1) and (10–1)/(−101), and the prismatic faces (021)/(0–2–1) for all nine supersaturations with the corresponding fixed solution temperatures. As expected, growth rates were generally found to increase with supersaturation with the growth rates of capping faces (101)/(−10–1), (−101)/(10–1) being observed to be much faster than that for the prismatic faces (021)/(0–2–1). This is consistent with the findings of Kitamura and Ishizu^30^ that the prismatic faces grow too slow to produce meaningful growth kinetics from their experiments, which is evidenced by the experimental run at σ = 0.28 (Table 1) of this study, and also the large discrepancy of the growth rate of the {021} face obtained from in-process β-LGA crystal images in crystallizers^16^ due to its much slower growth.

**Table 1:**
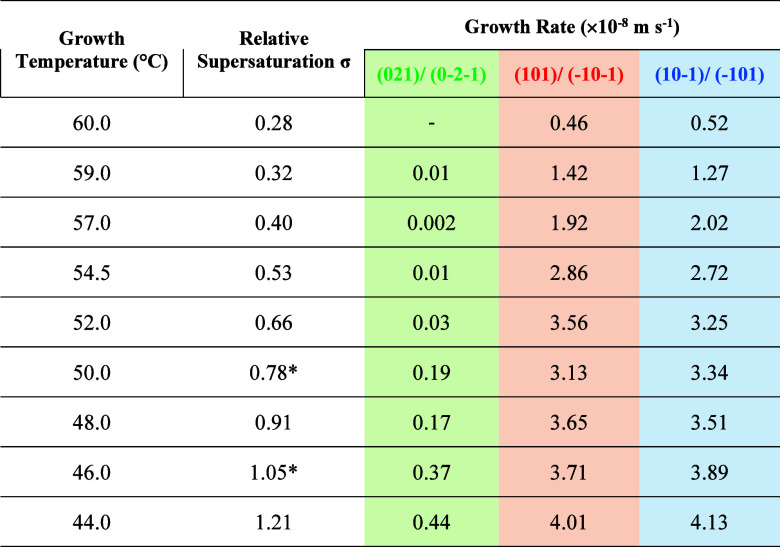
Facet Growth Rates of {101} Capping and {021} Prismatic Faces Under Different Supersaturations^a^

a Note that the facet growth rates at σ = 0.78* and 1.05* represent their corresponding mean values.

Table 2 shows a comparison between the growth rate measurement presented here with those from the literature.^11−13,15,16,21,30^ It can be seen that while the growth rates may differ between the different experimental setups or conditions, they broadly agree within the same order of magnitude. A big discrepancy of the growth rates for capping faces exists between the studies of Kitamura and Ishizu^21,30^ and others^11−13,15,16^ with the latter growing about 2.5–9.5 times faster than the former (Table 2) even in comparison with the result from an apparently similar flow cell setup.^15^ This might reflect, to a degree, the results from the low-resolution crystal images captured by video TV system.^21,30^ Overall, this study reveals that the growth rates of prismatic faces were consistently many times lower than those for the capping faces aligned with literature data (Table 2).

**Table 2:** Experimentally Measured Facet Growth Rates of β-Form LGA Crystals from the Literature and This Study^a^

		**growth rate (×10**^**–8**^ m s^–1^)		
reference	σ	capping faces {101}	prismatic faces {021}	data acquisition method
Kitamura and Ishizu^30^	0.48	0.37	N/A	microscope with video TV system
Kitamura and Ishizu^21^	0.48	0.46	N/A	microscope with video TV system
Ma et al.^13^	0.49	*3.52*	*0.71*	online in-process imaging system
Wang et al.^16^	0.49	*1.17*	*0.30*	online in-process imaging system
Ma and Wang^12^	0.49	*1.33*	*0.38*	online in-process imaging system
Ochsenbein et al.^15^	0.20	*1.91*	*0.07*	stereoscopic imaging with flow cell
Huo and Guan^11^	0.5	*1.76*	*0.81*	online imaging system
this study	0.28 – 1.21	0.46–4.13	0.002–0.44	in situ microscope with growth cell
	(0.40)	(1.92–2.02)	(0.002)	
	(0.53)	(2.72–2.86)	(0.01)	

a Note that the values in italics are the growth rates converted from measured lengths and widths to {101} capping and {021} prismatic faces for comparison purpose with the two supersaturation values (0.40 and 0.53) and their corresponding growth rates in parentheses being provided also for easy comparison.

### 4.3 Face-Specific Crystal Growth Kinetic Mechanisms

**Figure 7:**
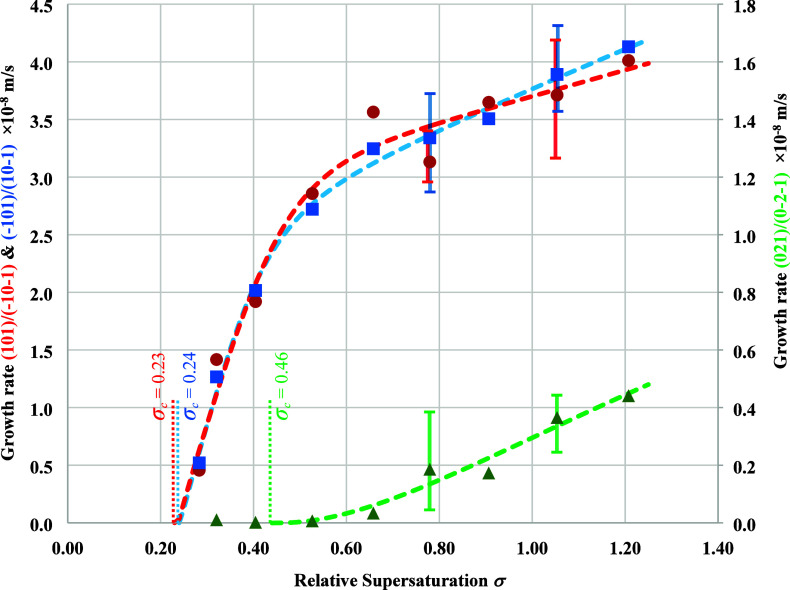
Measured growth rates (symbols) of β-LGA crystals of different faces and their best kinetic model fitting (dashed lines): red cycles and dashed line (B&S): (101)/(−10–1) faces; blue squares and dashed line (B&S): (10–1)/(−101) faces; green triangles and dashed line (BCF): (021)/(0–2–1) faces. The vertical dashed red, blue, and green lines indicate the critical relative supersaturations obtained from kinetics model fittings for the (101)/(−10–1) faces, (10–1)/(−101) faces, and (021)/(0–2–1) faces, respectively. For supersaturations of 0.78 and 1.05, the mean growth rates were used for the fittings with the minimum and maximum growth rates being plotted by error bars.

The fitting results for different faces using the crystal growth kinetics fitting model,^25^ including both growth resistances of MT and GSI, are listed in Table 3 with the best fitting models highlighted with bold and italic texts and shown in Figure 7. The best fitting model for capping faces (101)/(−10–1) and (10–1)/(−101) is the B&S model with the *R*^2^ being 0.97 and 0.99, respectively, for the two capping faces, although the power law fitting was found to have the similar high values of *R*^2^, but the unrealistic power value of ∼1.0 × 10^–14^ (Table 3) makes the fitting unreliable. For the prismatic faces (021)/(0–2–1), it was found that only the BCF model produced reasonably good fitting results with *R*^2^ = 0.97. Table 3 shows that at the average σ (= 0.75), for the {101} capping faces, the values of MT resistance (9.81 × 10^6^ and 9.18 × 10^6^) were found to be larger than those of the GSI resistance (5.24 × 10^6^ and 6.18 × 10^6^), consistent with the mass transfer of solute molecules from the bulk solution to the {101} capping faces being the growth limiting factor. However, for the {021} prismatic faces, the value of GSI resistance (1.74 × 10^8^) was found to be greater than that of the MT resistance (7.83 × 10^7^); consistent with the GSI being the main resistance to the growth of the {021} prismatic faces.

**Table 3:**
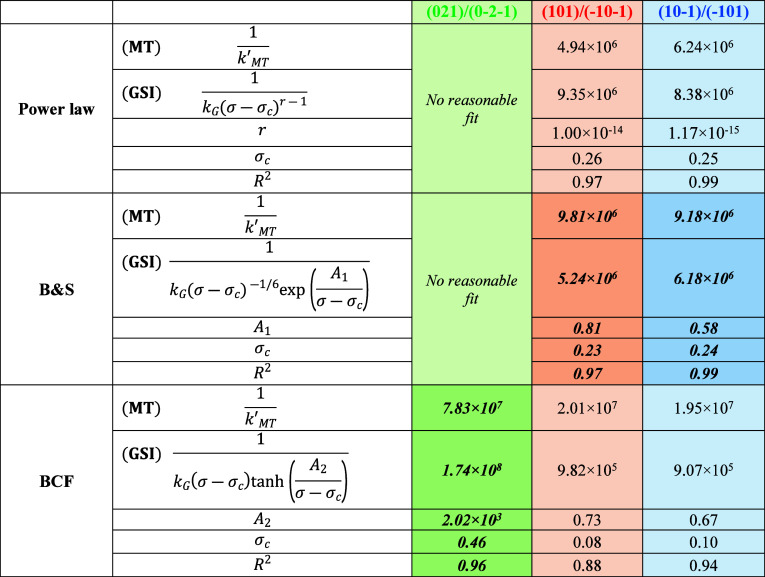
Parameters of Crystal Growth Kinetics from the Best Fitting of Experimental Growth Data with the Models (eqs 1–6)^a^

a Note that the data listed in this table are calculated using the fitting models with σ = 0.75 (the average value of the superstations studied in the present work). The bold and italic texts donate the typical best fits determined by the highest goodness-of-fit (*R*^2^)

The calculated resistance values on MT and GSI on facet growth rates under nine different supersaturations are given in Table 4. Within the relative supersaturation range studied (0.28–1.21), it can be seen that on the prismatic faces, (021)/(0–2–1), the resistance of GSI is generally greater than that of MT, which means that the GSI is the rate-limiting step. For the capping faces, the resistance of MT is greater than that of GSI, which indicates that the MT within bulk solution is the rate-limiting factor on these faces. However, at the higher supersaturations, the resistances due to both MT and GSI are roughly equivalent, with those two factors gradually playing a more balanced role in mediating the face-specific growth. This probably is a reflection of the higher solute concentration and hence higher viscosity leading to stronger MT resistance.

**Table 4:**
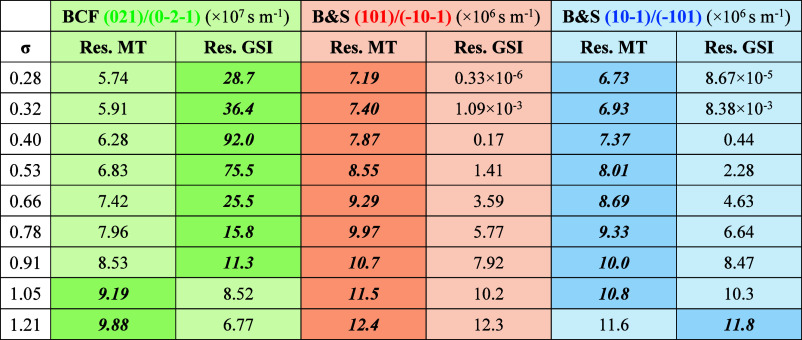
Resistances of Mass Transfer and Surface Integration on Different Crystal Faces Under Various Supersaturations^a^

a Note that the bold and italic values indicate the dominant resistances to growth.

### 4.4 Face-Specific Supersaturation Dead Zones

The critical relative supersaturation (*σ*_c_) or dead zone for crystallization, as defined as the range of 0–*σ*_c_ with no growth being observed as obtained from the model fittings, was found to be 0.23, 0.24, and 0.46 for the {101} and {−101} capping faces and the {021} prismatic faces, respectively, which are given in Figure 7 and Table 3.

The data highlights that a significantly higher supersaturation is needed to initiate growth on the prismatic faces compared to that on the capping faces consistent with a smaller cluster size for surface nucleation in the former case. These findings are in agreement with the recent morphological analysis by Turner et al.,^47^ who demonstrated that the prismatic faces have a much lower surface attachment energy when compared to the capping faces. Hence, this result is not unexpected.

Interestingly, the face-specific dead zone data suggest that below the *σ*_c_ of the {021} faces, growth should only occur on the capping faces.

## 5 Conclusions

The growth of both capping and prismatic faces of β-LGA was measured in situ using a temperature-controlled crystal growth cell with optical microscopy. An automatic sizing methodology using state-of-the-art machine learning-based computer vision techniques to segment the images was developed to quantify the facet crystal growth rates as a function of solution supersaturation, with their associated interface kinetic growth mechanisms being determined. Evaluation of the new methodology against existing manual and semi-automatic approaches demonstrates its equivalent accuracy in a much shorter time. It was found from the crystal growth kinetic data that the growth of the {101} capping faces was consistent with a Birth and Spread mechanism, while growth of the {021} prismatic faces was consistent with a Burton–Cabrera–Frank screw dislocation mechanism, with the former being in agreement with the available literature. Determination of rate-limiting kinetic parameters revealed growth of the {101} capping faces to be dominantly controlled by the MT of solute molecules from the bulk solution to the crystal surfaces, while the molecular integration process on the crystal surface generally limits the growth of the {021} prismatic faces, which is consistent with its elongated morphology. Both capping and prismatic faces were found to have dead zones associated with their facet growth, with the {021} prismatic faces being found to have larger (∼2 times) dead zone compared to the {101} capping faces. This suggests that the nucleation requires a higher supersaturation to initiate growth and concomitantly a smaller cluster size.

Further work is currently in progress to address the more challenging issue of directly measuring the growth of the crystals normal to the slowest growing faces {010}, i.e., in the vertical direction with respect to the optical axis of the microscope, by combining machine learning with molecular-based morphological modeling. Techniques are also under development to directly monitor the solute concentration during the crystal growth and, through this, be able to utilize the full set of temporal data acquired during the growth process. The outcomes from this work will be reported in due course. Overall, the face-based growth rates and growth mechanisms obtained in this study form part of an integrated digital design strategy encompassing morphological population balance modeling linked to hydrodynamic modeling of industrial scale crystallization.^4^

## Supporting Information Available

Linear fitting method for determining facet growth rate; typical sequences of crystal images of β-LGA growing from water; schematic of calculating normal distance between paired {021} prismatic faces; examination of experimental repeatability at σ = 0.78 and 1.05; and facet growth data of all nine experiments (PDF)

## Notes

Based on the presentation at the 22nd International Symposium on Industrial Crystallization (ISIC) and the 52nd Annual Meeting of the British Association for Crystal Growth (BACG).

## Abbreviations

*A*_1_, *A*_2_: thermodynamic parameters
*C*_e_: equilibrium concentration (solubility)
*G*: facet growth rate
*k*_G_: growth rate constant
mass transfer resistance
surface integration resistance
MW_s_: solute molecular weight
*r*: growth exponent
*ρ*_s_: solute density
σ: relative supersaturation
*σ*_c_: critical relative supersaturation
BCF: Burton–Cabrera–Frank model
B&S: Birth and Spread model
GSI: growth surface integration
LGA: l-glutamic acid
MT: mass transfer
RIG: rough interface growth model
SAM: segment anything model
VGG: visual geometry group
α-LGA, β-LGA: α-form LGA, β-form LGA
