# Supplementary material for: Automated Growth Rate Measurement of the Facet Surfaces of Single Crystals of the β-Form of l-Glutamic Acid Using Machine Learning Image Processing
Source: Cryst Growth Des. 2024 Apr 5;24(8):3277–88. doi: 10.1021/acs.cgd.3c01548 (PMC11036364; doi:10.1021/acs.cgd.3c01548)
Supplement: Supplementary file 1 — cg3c01548_si_001.pdf [file cg3c01548_si_001.pdf]

Supporting Information

Automated Growth Rate Measurement of the Facet Surfaces of Single Crystals of the β-Form L-Glutamic Acid Using Machine Learning Image Processing

Chen Jiang [1], Cai Y. Ma [1], Thomas A. Hazlehurst [2], Thomas P. Ilett [2], Alexander S. M. Jackson [1], David C. Hogg [2], Kevin J. Roberts [1,*]

[1] Centre for the Digital Design of Drug Products, School of Chemical and Process Engineering, University of Leeds, Leeds, LS2 9JT, UK

[2] School of Computing, University of Leeds, Leeds, LS2 9JT, UK

* Corresponding author: k.j.roberts@leeds.ac.uk

This supplementary material supports the main manuscript by providing further details of the following: Section S1 discusses about the linear fitting methods with **Figure S1** presenting a typical linear fitting for determining the face-based growth rate and **Table S1** listing the goodness-of-fitting (R^2) for all experimental runs. **Figure S2** shows a typical sequence of images of β-form l-glutamic acid (LGA) crystals growing from water with time at relative supersaturations of 0.28 and 1.05. Section S3 describes a method to calculate normal distances between the paired prismatic {021} faces with **Figure S3** showing the schematic of the method. Section S4 presents the repeated experimental data at σ = 0.78 and 1.05 to examine their repeatability, with **Figures S4 & S5** for experimental data and **Tables S2&S3** for their growth rates. **Figure S6** presents the lengths of facet growth as a function of growth time for all nine experiments (nine different supersaturations).

S1. Linear Fitting for Determining Growth Rate

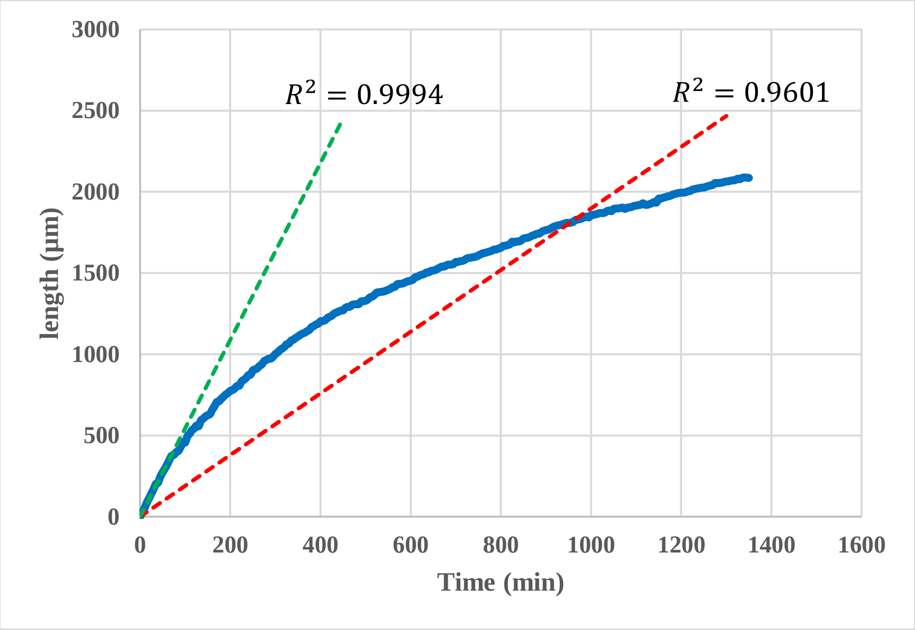

Figure S1. Determination of growth rate from relative length (to the first image) against relative time. The linear fitting is applied, and the gradient of the fitted line represents the growth rate. The red dotted line fits all data within the time range. The decrease of growth rate over time would cause poor fitting results, hence the R^2 of fitting being relatively lower.

The decrease of growth rate is likely caused by the decrease of supersaturation within the cuvette during crystal growth. It is preferred to only use the data at the beginning of the growth, hence the initial supersaturation still being valid. For example, the green dotted line fits only the first 100 min of data with very decent fitting results ($R^2 \approx 1$). Therefore, the gradient of the green line is used as the growth rate. Over all sets of the experimental data, the data from the first 60 – 810 min (about 20 - 180 data points) as shown in **Table S1** are included for the linear fitting. As shown in **Table S1**, all of the linear fittings for the {101} capping faces have very high R^2 (> 0.99), whilst most of the goodness of fittings for the {021} prismatic faces are

reasonably satisfactory with some runs at lower supersaturations being found to have lower R^2. This most possibly results from the very low growth rate of the prismatic faces at low supersaturations, hence highly scattered data measured, even more at the lowest σ (= 0.28) there is no growth rate for the prismatic faces at all as evidenced in literature [1].

Table S1. The R^2 of linear growth rate fitting with the time for the early stage and the corresponding data points. Note that the run #6 (σ = 0.78) is an outlier.

σ	Run #	Time (min)	Number of Images	R^2 (021)/(0-2-1)	R^2 (101)/(-10-1)	R^2 (10-1)/(-101)
0.28	1	90	18	-	0.989	0.997
0.32	1	480	48	0.876	0.991	0.990
0.40	1	670	134	0.598	0.994	0.990
0.53	1	310	62	0.433	0.998	0.999
0.66	1	645	129	0.949	0.992	0.996
0.78	1	345	69	0.891	0.993	0.994
	2	105	21	0.910	0.997	0.999
	3	60	12	0.997	0.997	0.998
	4	60	12	0.997	0.996	0.998
	5	200	40	0.969	0.992	0.989
	6	210	42	0.980	0.997	0.998
0.91	1	535	107	0.983	0.997	0.997
1.05	1	355	71	0.984	0.992	0.995
	2	250	50	0.997	0.991	0.992
	3	610	122	0.991	0.995	0.993
	4	810	162	0.976	0.982	0.991
	5	225	45	0.993	0.991	0.991
1.21	1	110	22	0.993	0.996	0.991

S2. Typical Sequence of Crystal Images Captured

σ = 0.28

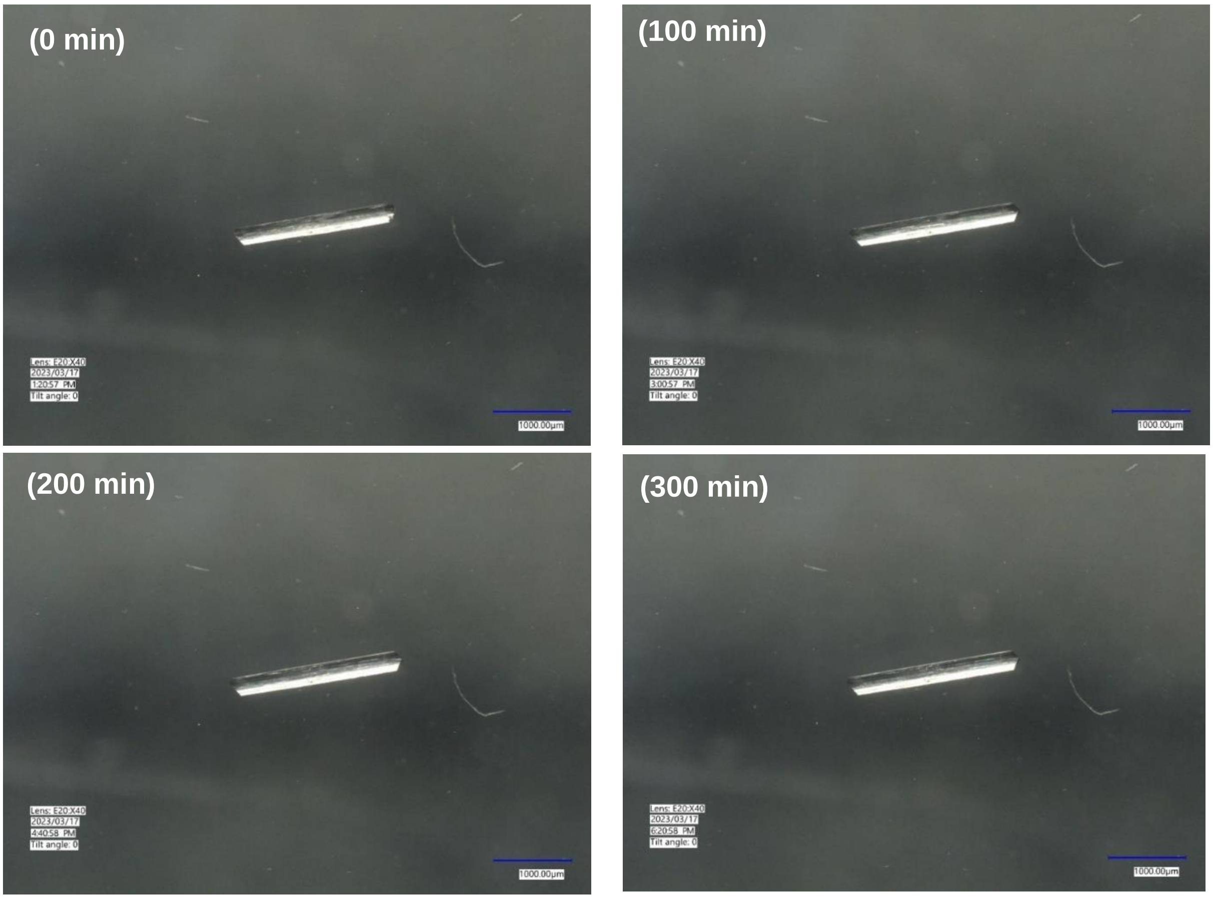

σ = 1.05

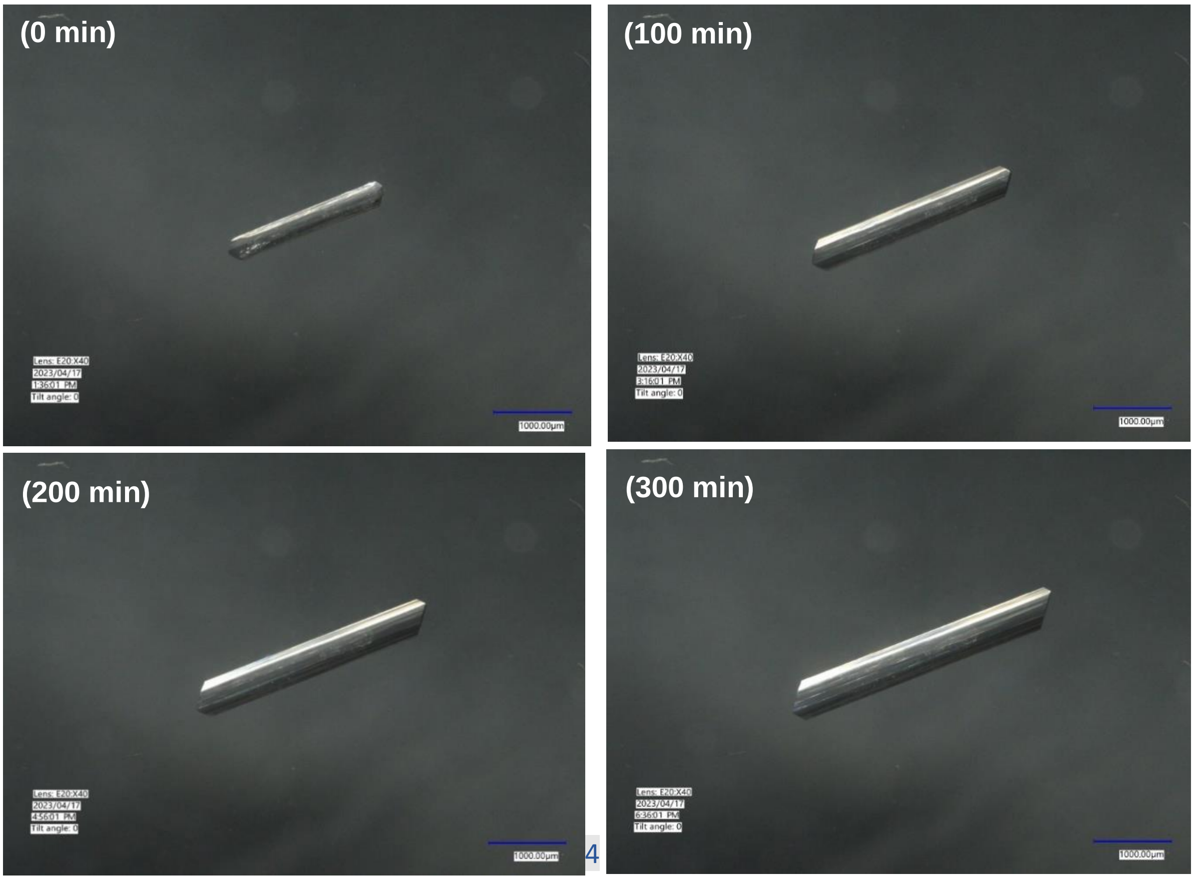

Figure S2. Typical sequence of images of beta-form LGA crystals growing in water with time at relative supersaturations of 0.28 and 1.05.

S3. Calculation of Normal Distance between Paired {021}

As shown in Figure S3 (SI), the normal distance between the paired (021) and (0-2-1) faces were calculated based on the projected width, *w*, and the angle, *θ*, between (021) and (02-1) faces by the following equation (S1):

$$d_{(021)/(0-2-1)} = w\, sin\frac{\theta}{2} \qquad \text{(S1)}$$

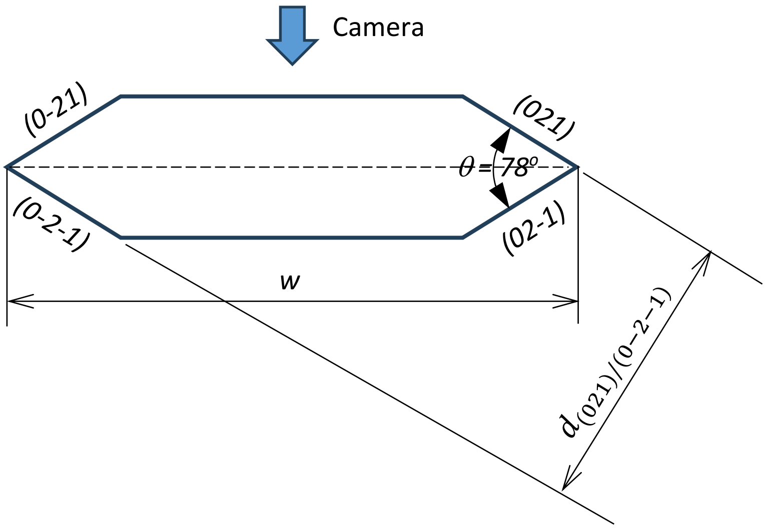

Figure S3. Schematic of facet normal distance calculation of the prismatic {021} faces.

S4. Repeatability of Experimental Results

The growth rates for the experimental data at σ = 0.78 and 1.05 with six and five repeats are shown in **Tables S2 & S3**, respectively. At σ = 0.78, as shown in **Figure S4**, the run #6 coloured as red was found to have much faster growth in all three face directions. Further examinations found that the corresponding growth rates are about 2 times that of the other 5 runs, hence treating as an outlier. The mean growth rates (without the outlier) were found to be 3.13×10^{-8}, 3.34×10^{-8} and 0.19×10^{-8} m/s for faces (101)/(-10-1), (10-1)/(-101) and (021)/(0-2-1) with their corresponding standard deviations being 0.27×10^{-8}, 0.47×10^{-8} and 0.19×10^{-8} m/s (**Table S2**). At σ = 1.05, the growth data is given in **Figure S5**. The mean growth rates were found to be 3.71×10^{-8}, 3.89×10^{-8} and 0.37×10^{-8} m/s for faces (101)/(-10-1), (10-1)/(-101) and (021)/(0-2-1) with their corresponding standard deviations being 0.55×10^{-8}, 0.42×10^{-8} and 0.12×10^{-8} m/s (**Table S3**). These standard deviations are compatible with the growth rate measurements of ibuprofen growing in different solvents using a growth cell [2]. The discrepancies might result from the variations of seeds sizes (for σ = 0.78, 1699 – 2815 μm for faces (101)/(-10-1), 1693 – 2819 μm for faces (10-1)/(-101) and 148 – 249 μm for faces (021)/(0-2-1); for σ = 1.05, 1395 – 1759 μm for faces (101)/(-10-1), 1375 – 1803 μm for faces (10-1)/(-101) and 130-308 μm

for faces (021)/(0-2-1)), and the possible growth rate dispersion. The larger discrepancy for the faces (021)/(0-2-1) might also be caused by the very slow growth as mentioned in literature [1], hence more scattering data.

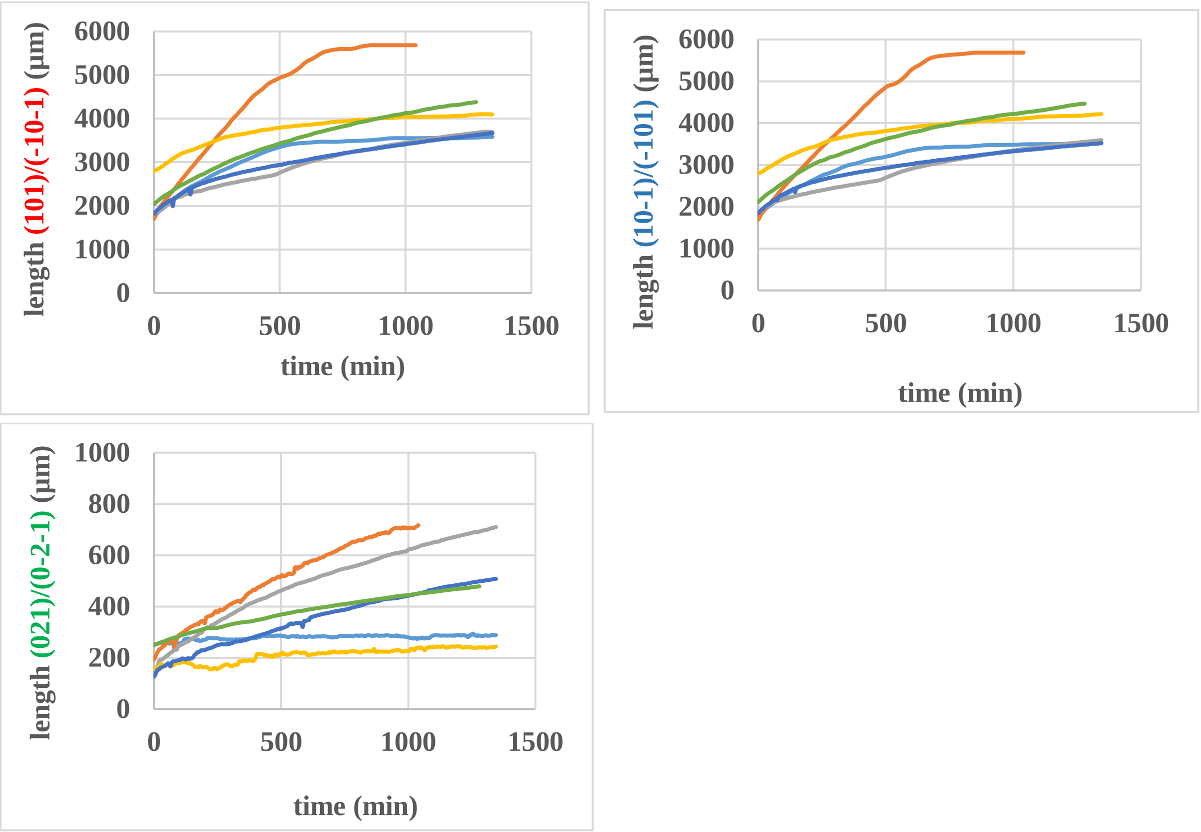

Figure S4. Lengths of capping and prismatic faces vs time at σ = 0.78 from six repeated experiments. Note that the experimental run (coloured as red) can be treated as an outlier due to its much faster growth (about 2 times of growth rate compared with other five runs).

Table S2. Facet growth rates measured from the repeated experiments at σ = 0.78. The colour of each run matches the corresponding colour as shown in **Figure S4**. Run #6 is an outlier which was not used for calculating the mean growth rate.

**Run #**	**Growth rates (×10^{-8} m s^{-1})**		
	**(021)/(0-2-1)**	**(101)/(-10-1)**	**(10-1)/(-101)**
1	0.04	3.40	3.27
2	0.14	2.96	2.87
3	0.38	3.06	3.50
4	0.18	3.11	3.72
5	0.30	3.33	3.41
6	0.45	6.62	6.14
Mean	0.19	3.13	3.34
Standard deviation	0.19	0.27	0.47

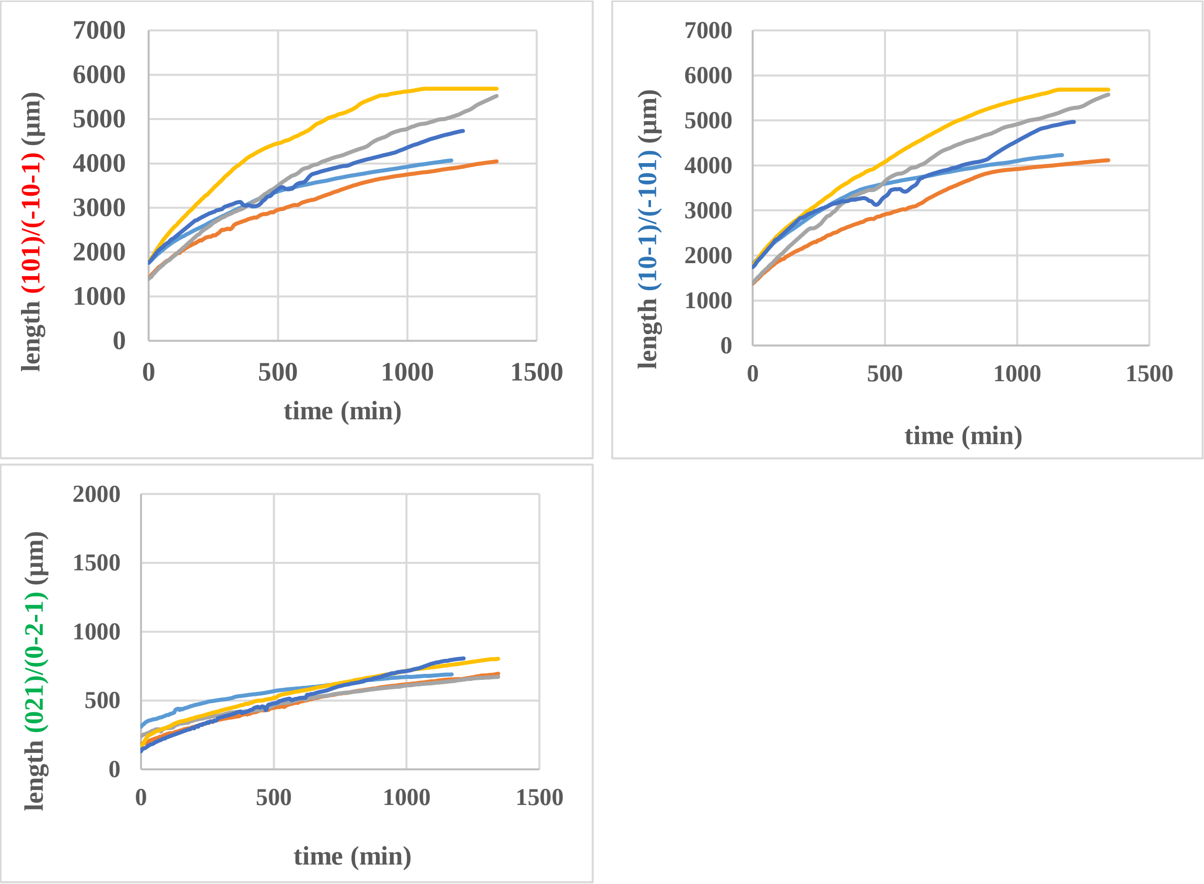

Figure S5. Lengths of capping and prismatic faces vs time at σ = 1.05 from six repeated experiments.

Table S3. Facet growth rates measured from the repeated experiments at $\sigma = 1.05$. The colour of each run matches the corresponding colour as shown in **Figure S5**.

Run #	Growth rates (×10^{-8} m s^{-1})		
	(021)/(0-2-1)	(101)/(-10-1)	(10-1)/(-101)
1	0.44	3.16	3.87
2	0.34	3.70	3.57
3	0.25	3.63	3.92
4	0.36	4.19	3.78
5	0.44	3.88	4.31
Mean	0.37	3.71	3.89
Standard deviation	0.12	0.55	0.42

S5. Crystal Growth of Capping and Prismatic Faces

a) σ = 0.28

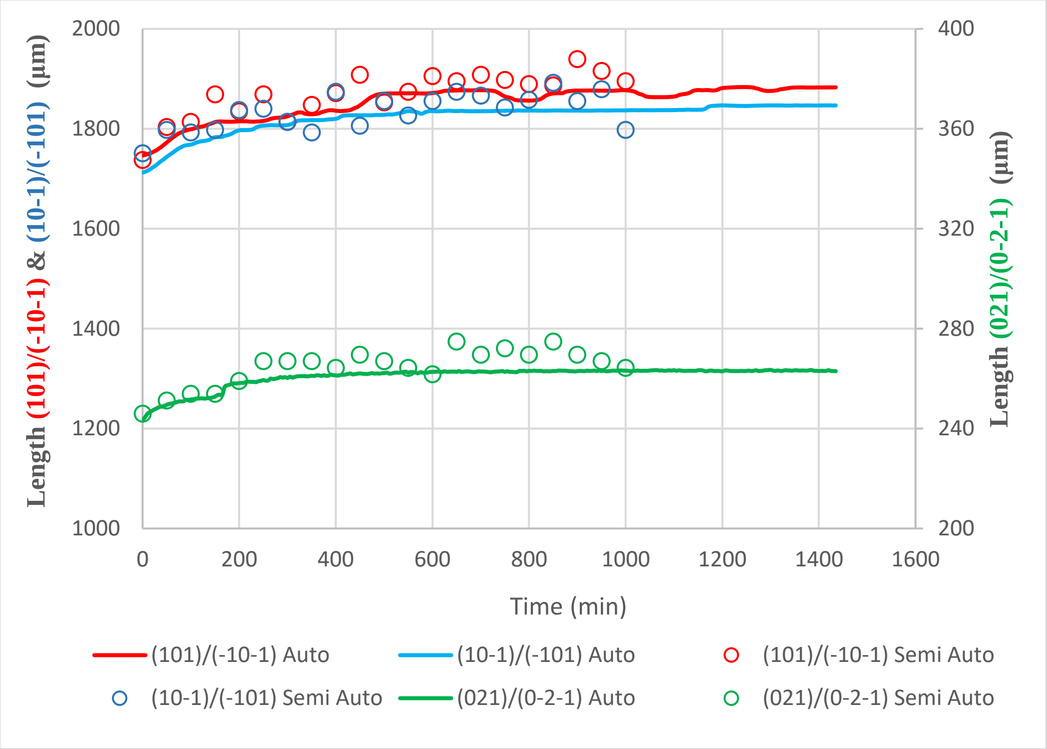

b) σ = 0.32

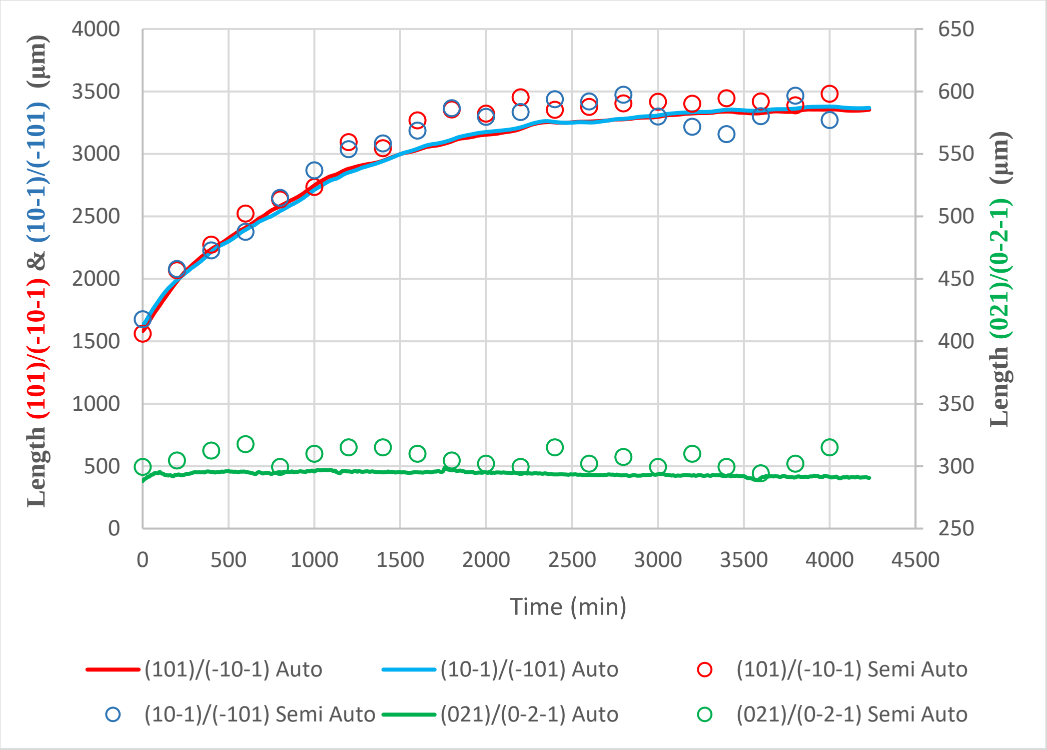

c) σ = 0.40

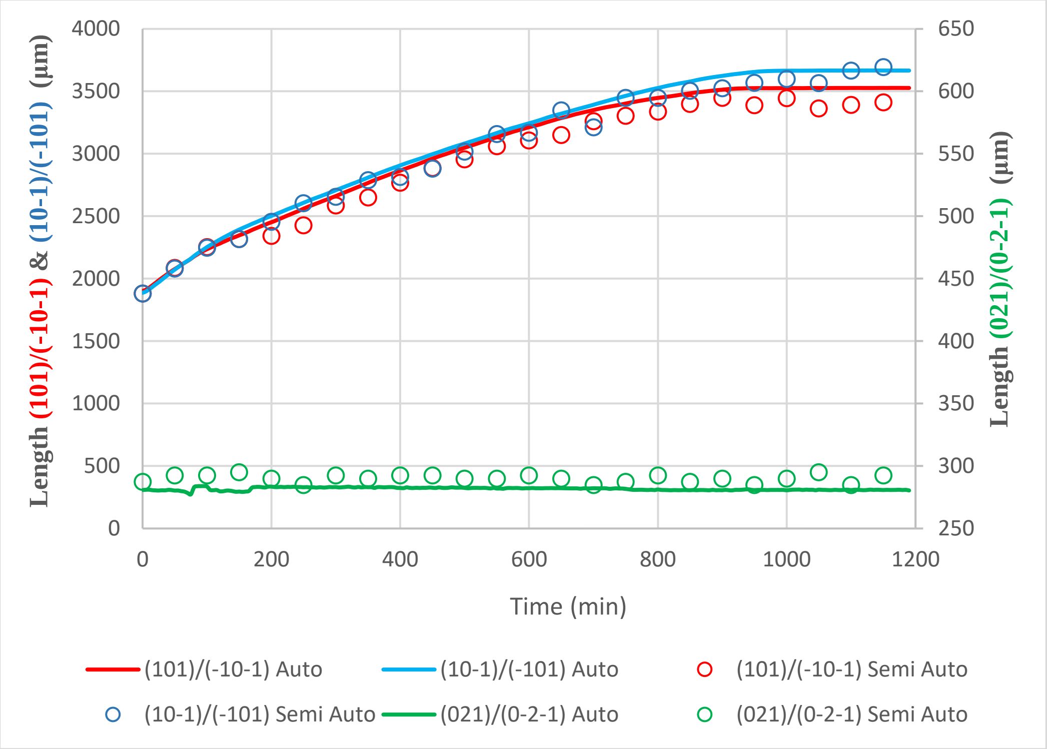

Length (101)/(-10-1) & (10-1)/(-101) (μm)
Length (021)/(0-2-1) (μm)
Time (min)
4000
3500
3000
2500
2000
1500
1000
500
0
650
600
550
500
450
400
350
300
250
0
200
400
600
800
1000
1200
(101)/(-10-1) Auto
(10-1)/(-101) Auto
(101)/(-10-1) Semi Auto
(10-1)/(-101) Semi Auto
(021)/(0-2-1) Auto
(021)/(0-2-1) Semi Auto

d) σ = 0.53

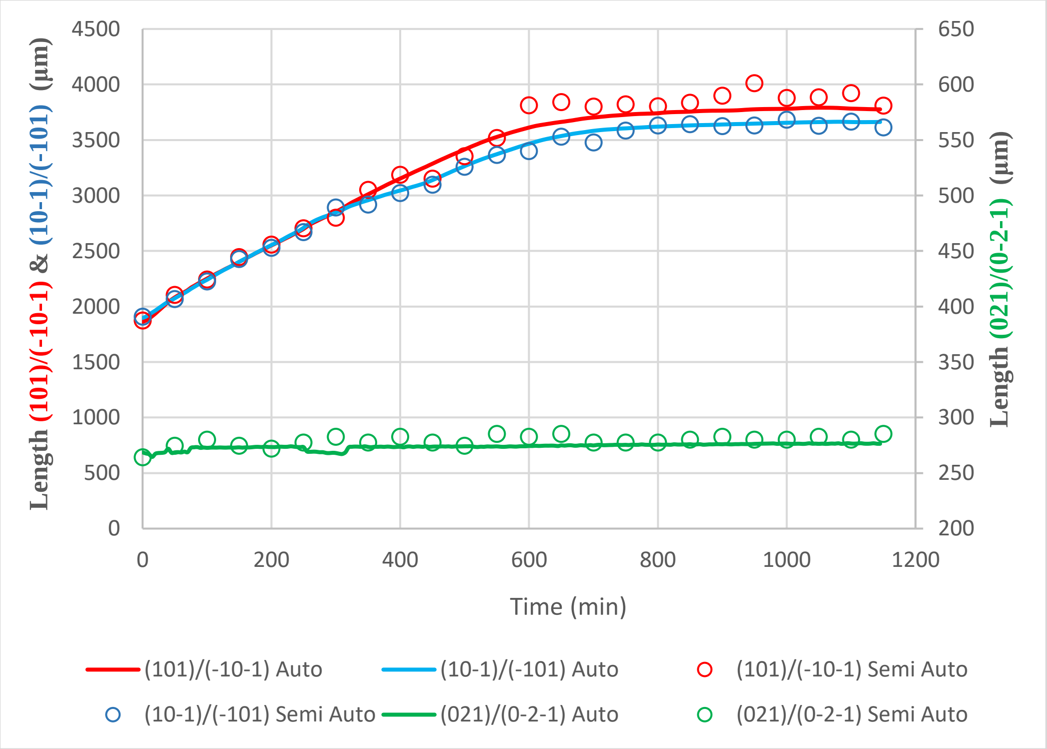

Length (101)/(-10-1) & (10-1)/(-101) (μm)
Length (021)/(0-2-1) (μm)
Time (min)
4500
4000
3500
3000
2500
2000
1500
1000
500
0
650
600
550
500
450
400
350
300
250
200
0
200
400
600
800
1000
1200
(101)/(-10-1) Auto
(10-1)/(-101) Auto
(101)/(-10-1) Semi Auto
(10-1)/(-101) Semi Auto
(021)/(0-2-1) Auto
(021)/(0-2-1) Semi Auto

e) σ = 0.66

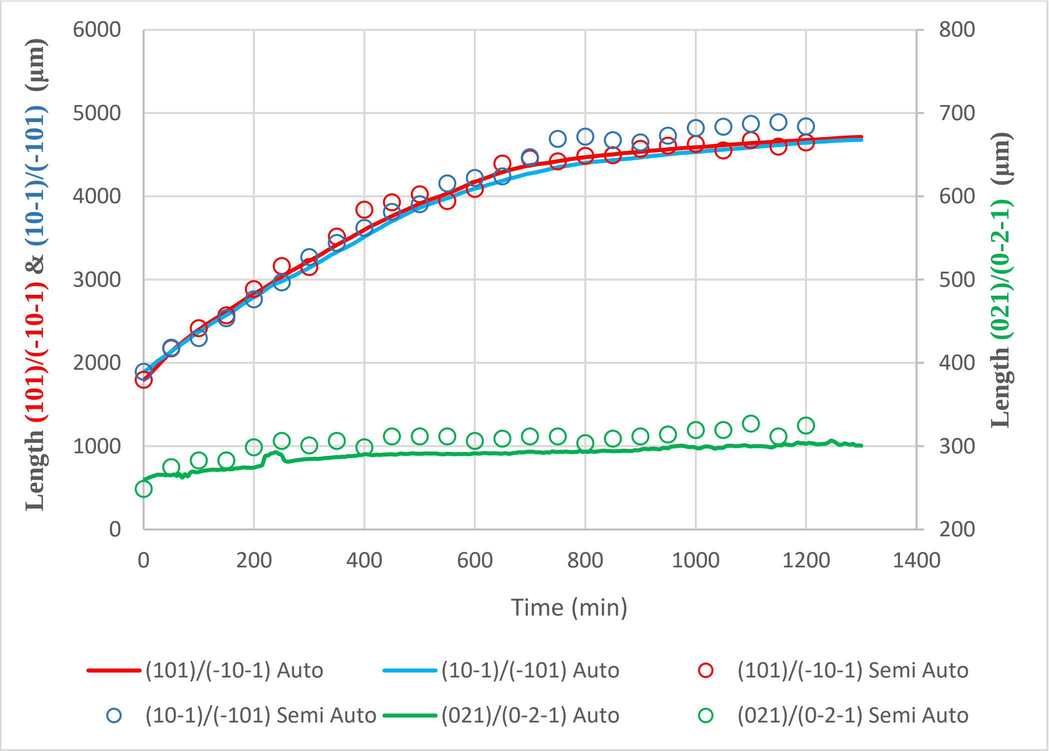

f) σ = 0.78 (representative run #1, **Table S2**)

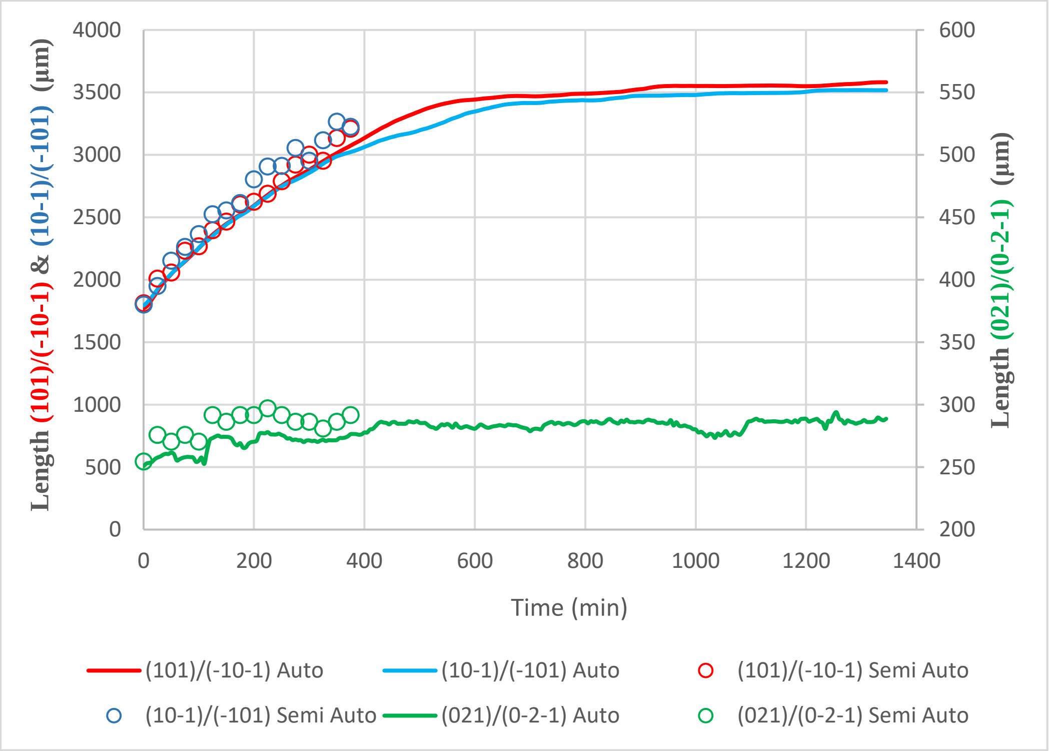

g) σ = 0.91

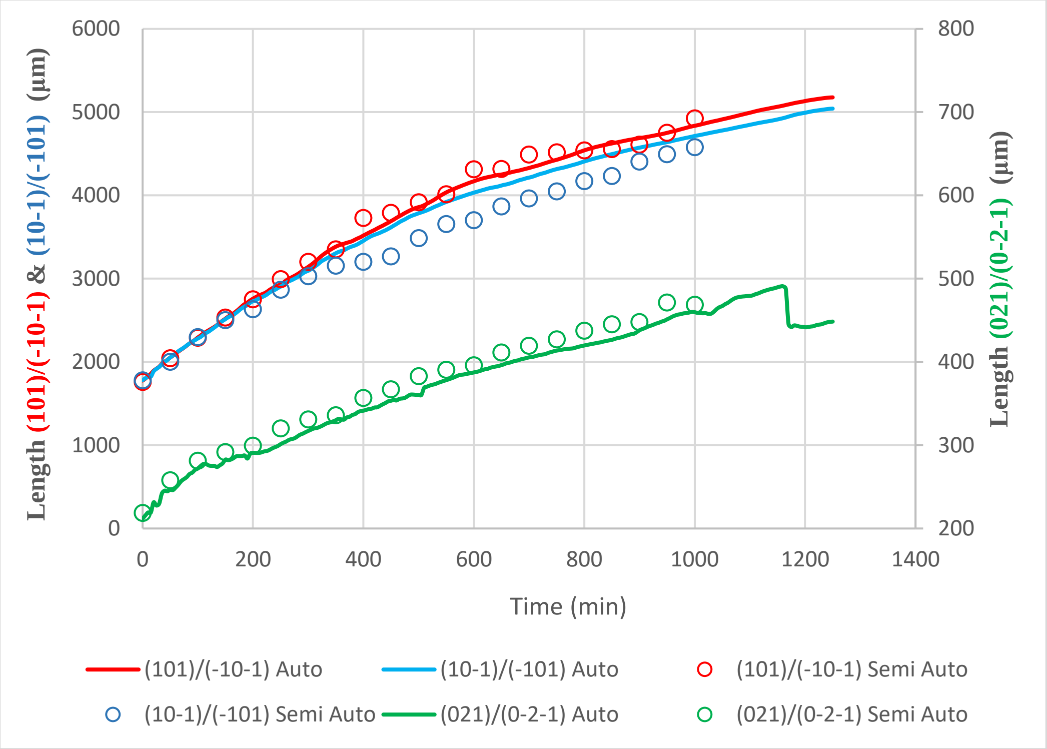

h) σ = 1.05 (representative run #1, **Table S2**)

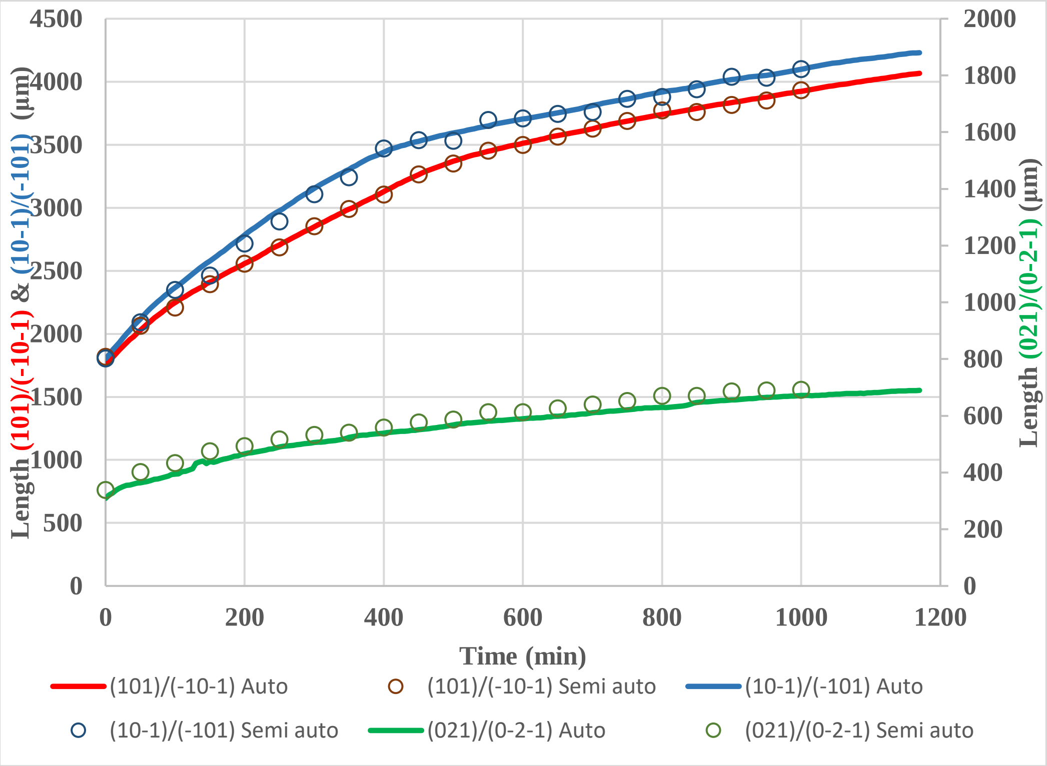

i) σ = 1.21

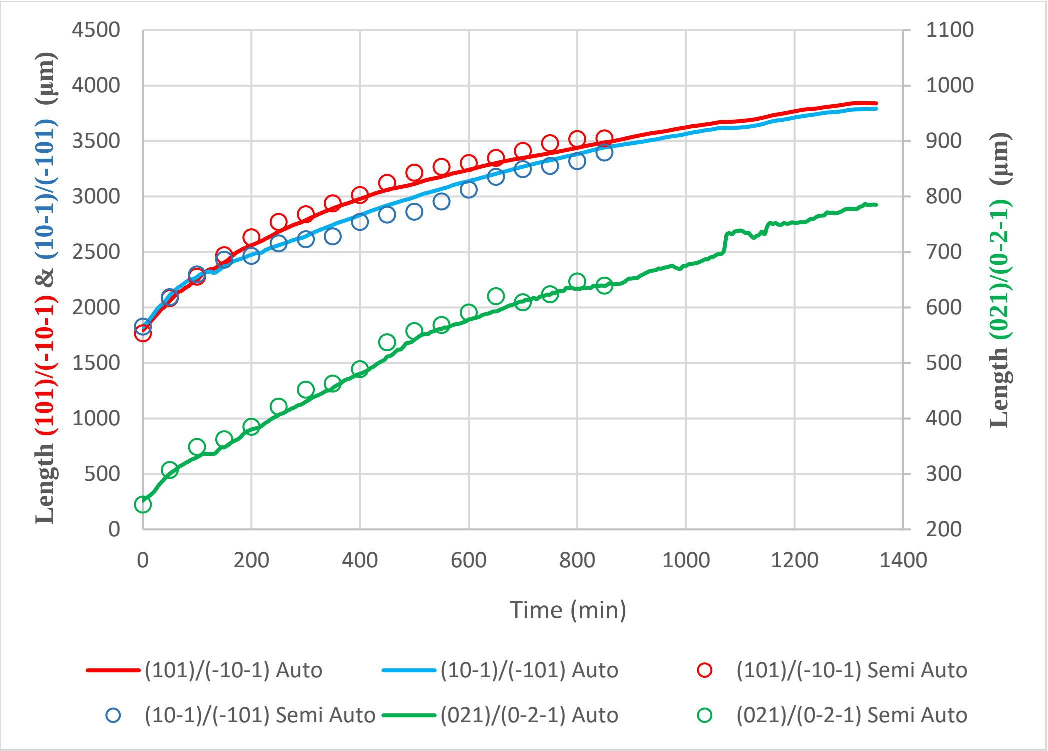

Figure S6. Normal distances (lengths) between paired crystal faces during crystal growth in a growth cell under nine different supersaturations, using the semi-automatic (o) and automatic sizing (—) methods (Red – (101)/(-10-1) faces; Blue – (10-1)/(-101) faces; Green – (021)/(0-2-1) faces).

References

(1) Kitamura, M.; Ishizu, T. Kinetic effect of L-phenylalanine on growth process of L-glutamic acid polymorph. *Journal of Crystal Growth* **1998**, *192*, 225-235. Kitamura, M.; Ishizu, T. Growth kinetics and morphological change of polymorphs of L-glutamic acid. *J. Cryst. Growth* **2000**, *209*, 138-145.

(2) Nguyen, T. T. H.; Hammond, R. B.; Roberts, K. J.; Marziano, I.; Nichols, G. Precision measurement of the growth rate and mechanism of ibuprofen {001} and {011} as a function of crystallization environment. *CrystEngComm* **2014**, *16* (21), 4568-4586. DOI: 10.1039/c4ce00097h.
